# Twentieth-century emergence of antimicrobial resistant human- and bovine-associated *Salmonella enterica* serotype Typhimurium lineages in New York State

**DOI:** 10.1038/s41598-020-71344-9

**Published:** 2020-09-02

**Authors:** Laura M. Carroll, Jana S. Huisman, Martin Wiedmann

**Affiliations:** 1 Department of Food Science, Cornell University, Ithaca, NY USA; 2 Department of Environmental Systems Science, ETH Zurich, Zurich, Switzerland; 3 Swiss Institute of Bioinformatics, Lausanne, Switzerland

**Keywords:** Antimicrobial resistance, Bacterial evolution, Bacterial genomics

## Abstract

*Salmonella enterica* serotype Typhimurium (*S.* Typhimurium) boasts a broad host range and can be transmitted between livestock and humans. While members of this serotype can acquire resistance to antimicrobials, the temporal dynamics of this acquisition is not well understood. Using New York State (NYS) and its dairy cattle farms as a model system, 87 *S.* Typhimurium strains isolated from 1999 to 2016 from either human clinical or bovine-associated sources in NYS were characterized using whole-genome sequencing. More than 91% of isolates were classified into one of four major lineages, two of which were largely susceptible to antimicrobials but showed sporadic antimicrobial resistance (AMR) gene acquisition, and two that were largely multidrug-resistant (MDR). All four lineages clustered by presence and absence of elements in the pan-genome. The two MDR lineages, one of which resembled *S.* Typhimurium DT104, were predicted to have emerged circa 1960 and 1972. The two largely susceptible lineages emerged earlier, but showcased sporadic AMR determinant acquisition largely after 1960, including acquisition of cephalosporin resistance-conferring genes after 1985. These results confine the majority of AMR acquisition events in NYS *S.* Typhimurium to the twentieth century, largely within the era of antibiotic usage.

## Introduction

*Salmonella enterica* subsp. *enterica* serotype Typhimurium (*S.* Typhimurium) consistently ranks as one of the serotypes most commonly isolated from human clinical cases in the United States; in 2016, it was reported as being responsible for 4,581 culture-confirmed human infections in the U.S. alone^1^. Additionally, *S.* Typhimurium is capable of infecting a broad range of hosts: it is frequently isolated not only from humans, but from animals as well, including livestock, rodents, and birds^2–4^.

Within the serotype, numerous *S.* Typhimurium lineages have been identified, several of which are notable due to their propensity for resistance to antimicrobials^5^. Many of these lineages have been assigned using phage typing, a practice by which *S.* Typhimurium can be differentiated based on its susceptibility to lysis by phages of varying specificity^6^. Host range can vary within phage type, as some phage types (e.g. DT104, DT204, DT49) are commonly associated with epidemics among livestock and humans^2,6,7^, while others exhibit a narrower host range (e.g. DT2 and DT99, which are highly virulent in pigeons)^6–9^. Additionally, a number of *S.* Typhimurium lineages are often associated with distinct antimicrobial resistance (AMR) profiles; for example, *S.* Typhimurium DT104 is often characterized by its resistance to ampicillin, chloramphenicol, streptomycin, sulfonamides, and tetracycline (ACSSuT), although AMR profiles within a lineage can vary^10–13^.

While whole-genome sequencing (WGS) is being increasingly employed to characterize *S.* Typhimurium from diverse sources at high resolution, the bulk of the effort has focused on characterizing distinct lineages that have been responsible for human epidemics (e.g. DT104)^12,14^. Furthermore, the temporal dynamics of AMR determinant acquisition in the serotype, as well as their loss, is not well understood. Here, we employ WGS to characterize *S.* Typhimurium isolated from human clinical, bovine, and bovine farm environmental sources in New York State (NYS) over an 18-year period. We offer estimates as to when various *S.* Typhimurium lineages of clinical importance emerged in NYS, including multidrug-resistant (MDR) lineages, and, using a parsimonious approach, characterize the temporal acquisition and loss of AMR determinants in the serotype.

## Results

### Human- and bovine-associated New York State *S. *Typhimurium are largely represented by four major lineages

**Figure 1:**
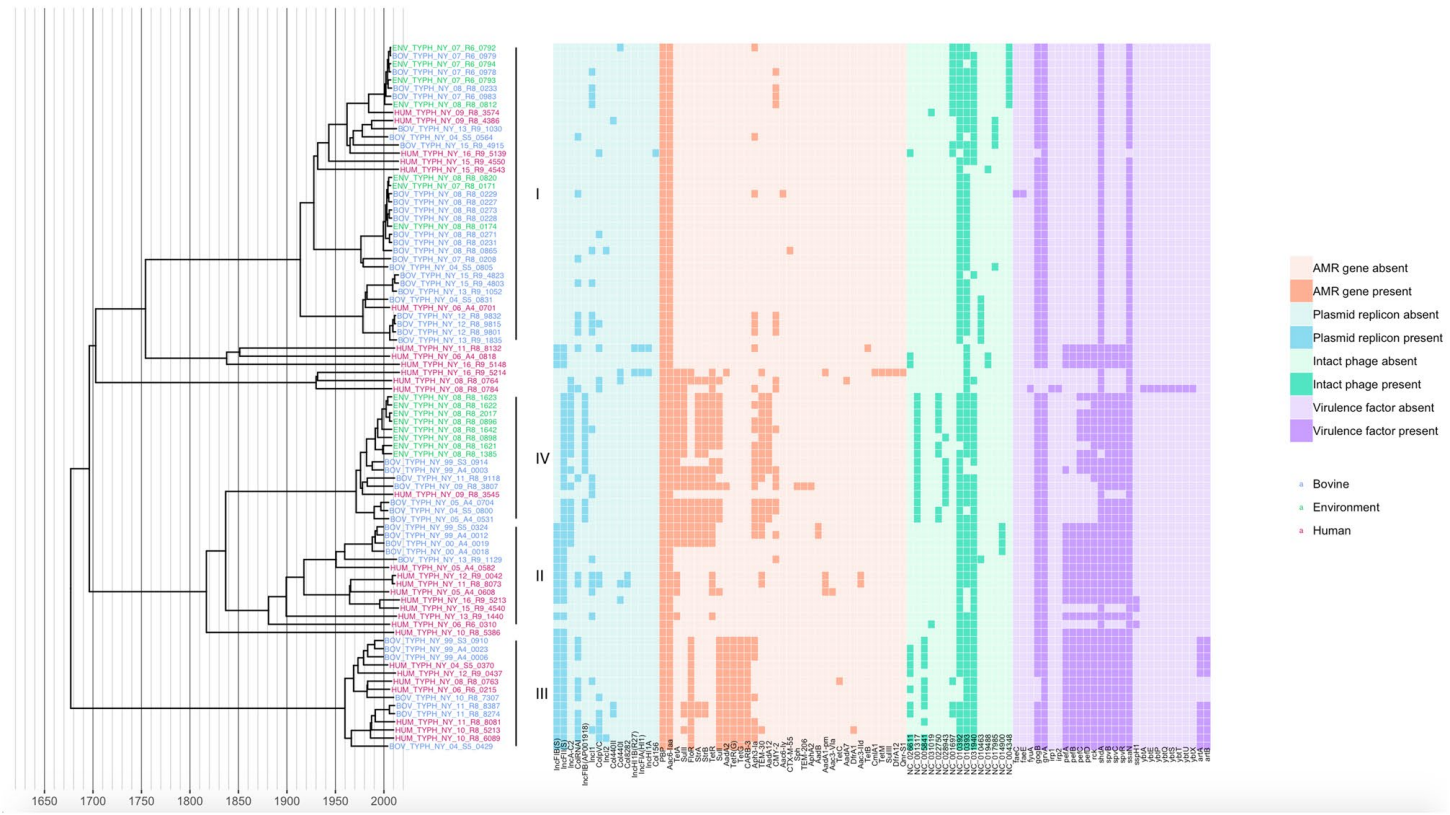
Maximum clade credibility phylogeny of 87 *S.* Typhimurium isolates from New York State with their corresponding (i) plasmid replicon, (ii) antimicrobial resistance (AMR) gene, (iii) intact phage, and (iv) virulence factor presence/absence profiles (displayed in the heatmap to the right of the phylogeny). Strains were isolated from bovine, bovine farm environmental, or human clinical sources (blue, green, and magenta tip labels, respectively) from 1999 to 2016. The time scale in years is plotted along the x-axis of the phylogeny, and lineages selected based on AMR gene clustering and rhierbaps are denoted by black clade labels I, II, III, and IV. Virulence factors present in all 87 genomes have been omitted from the heatmap to improve readability (see Supplementary Table S3).

Four major, well-supported lineages (posterior probabilities > 0.999) were present among the 87 *S.* Typhimurium isolated from bovine, bovine farm environmental, and human clinical sources (Fig. 1 and Supplementary Table S1; to view the phylogeny with posterior probabilities and node height 95% highest posterior density [HPD] intervals, see https://github.com/lmc297/NYS_Typhimurium_2018). Two of these lineages were largely antimicrobial-susceptible, with sporadic introduction of AMR determinants (Lineage I, n = 37; Lineage II, n = 13), while the other two major lineages were largely MDR (Lineage III, n = 14; Lineage IV, n = 16) (Fig. 1). These four lineages were consistently represented within the larger U.S. bovine and human *S.* Typhimurium phylogeny (Supplementary Figure S1), which was divided into six major lineages representing over 99% of U.S. *S.* Typhimurium (using the level 1 cluster assignments reported by rhierbaps; Supplementary Figure S2). Specifically, 86 of the 87 NYS *S.* Typhimurium genomes could be placed into one of the six major U.S. *S.* Typhimurium lineages, with each of the six major lineages represented by the NYS *S.* Typhimurium characterized in this study (Supplementary Figures S1 and S2).

Based on SNPs detected in the 87 NYS *S.* Typhimurium isolates from human clinical, bovine, and bovine farm environmental sources, the effective population size of *S.* Typhimurium in New York State reached its peak just prior to 2000 (Supplementary Figure S3). A mean clock rate of 3.267 × 10^−7^ substitutions/site/year (95% HPD [1.515 × 10^−7^, 5.181 × 10^−7^]) was estimated across all 87 *S.* Typhimurium lineages, which is within the range of clock rate estimates previously obtained for lineages within the serotype^12,15^.

### The pan-genome of *S. *Typhimurium displays lineage-dependent sub-structure

**Figure 2:**
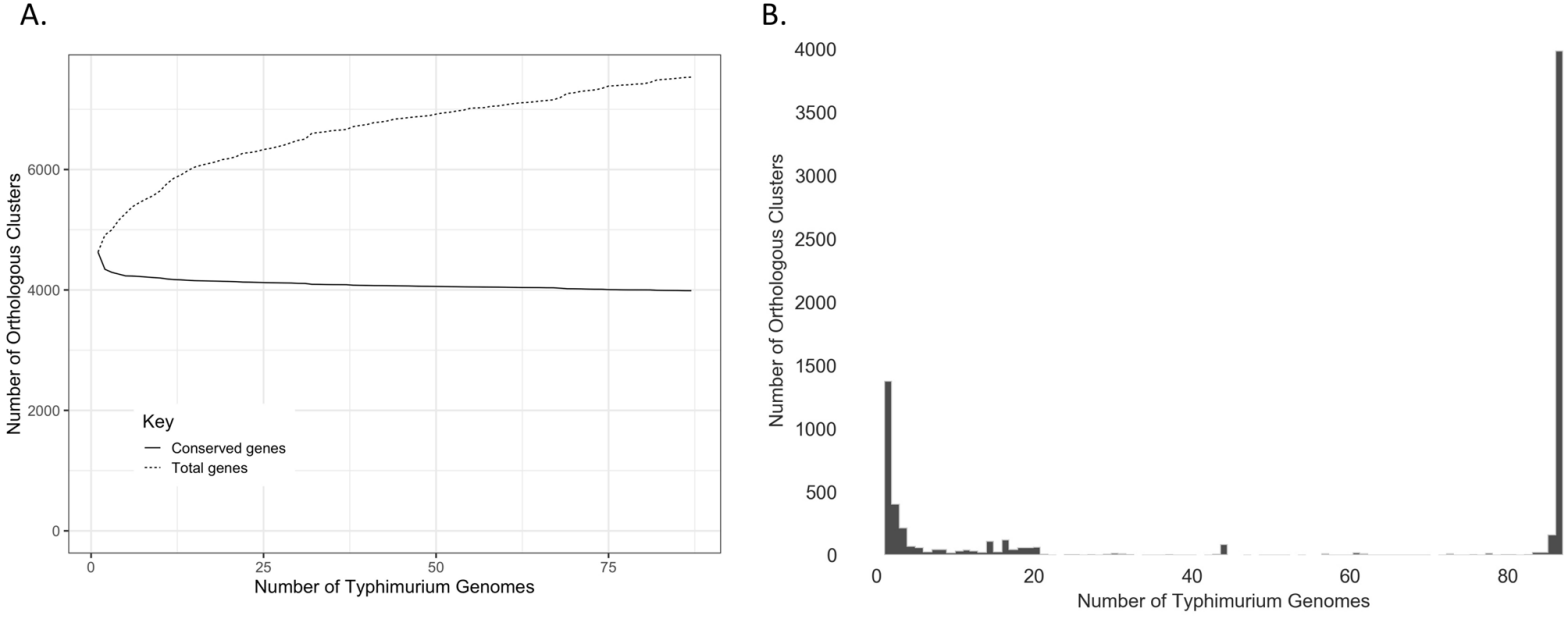
Pan-genome of 87 bovine- and human-associated *S.* Typhimurium isolates from New York State, 1999–2016. All plots were constructed using Roary version 3.11.0 and associated scripts. Panels display (**A**) the number of total (dotted line) and conserved (solid line) orthologous clusters detected in all 87 isolates as genomes were added randomly (constructed using Roary’s create_pan_genome_plots.R script), and (**B**) the number of orthologous clusters (y-axis) detected per number of genomes (x-axis; constructed using Roary’s roary_plots.py script).

**Figure 3:**
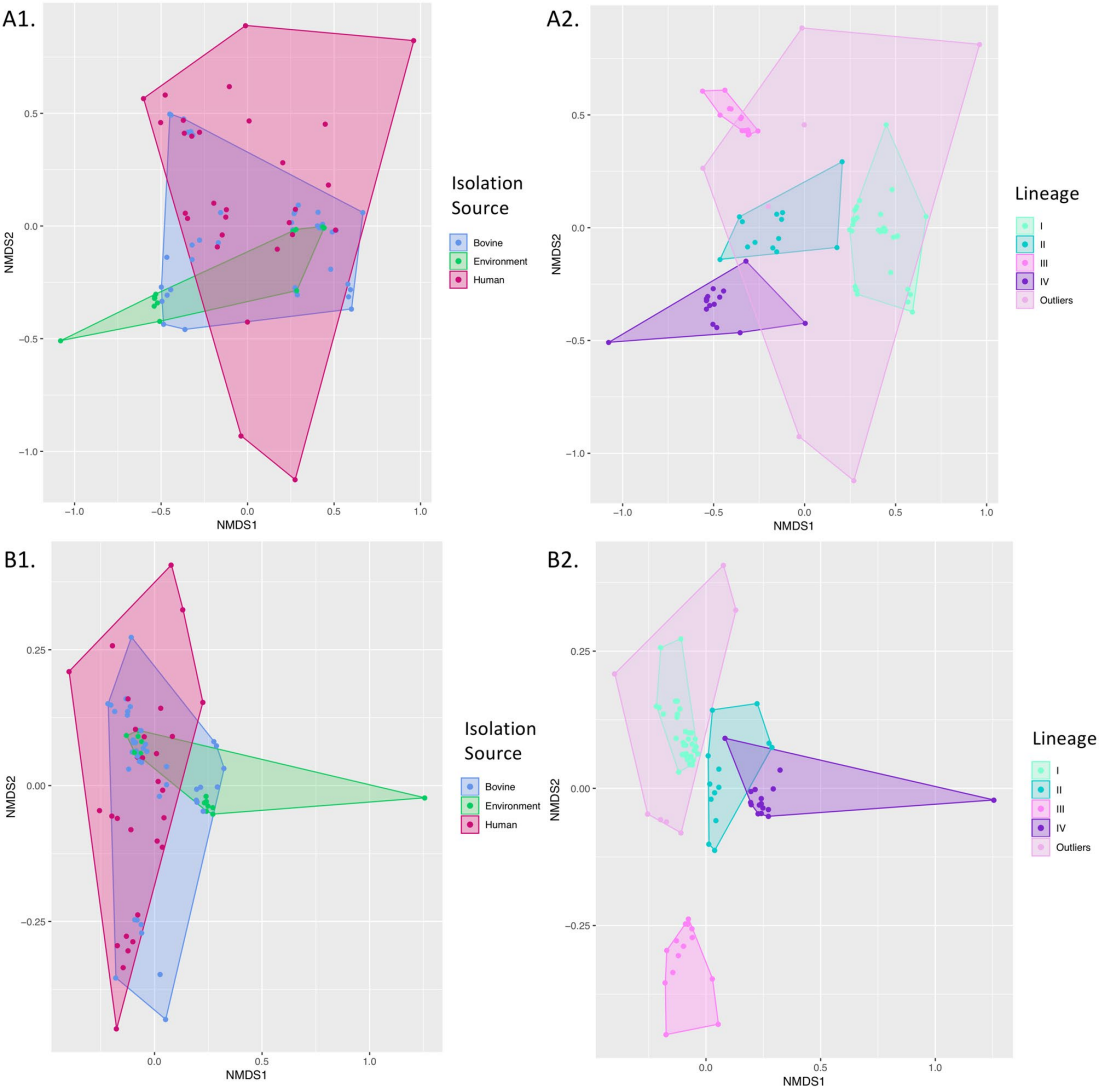
Non-metric multidimensional scaling (NMDS) plots of 87 *S.* Typhimurium genomes constructed using a Jaccard distance metric and presence/absence profiles of (**A**) orthologous gene clusters output by Roary version 3.11.0 and (**B**) gene ontology (GO) terms. Points represent isolates, while shaded regions and convex hulls correspond to (1) isolation source (bovine, bovine farm environment, or human clinical) and (2) lineage (I–IV, and “Outliers”, which encompasses the seven human clinical genomes that were not placed into one of the four major NYS *S.* Typhimurium lineages).

Of the 7,535 orthologous clusters detected among the 87 *S.* Typhimurium isolates, 3,988 (52.9%) represented core genes detected in all genomes (Fig. 2). Much in the same way that each of the four major lineages appeared to cluster by AMR profile, each of the four lineages clustered by presence/absence of pan-genome orthologous clusters, as well as presence/absence of Gene Ontology (GO) terms derived from all orthologous clusters (pairwise ANOSIM and PERMANOVA *P* < 0.05 after a Bonferroni correction; Fig. 3 and Supplementary Figure S4). Each of the four major *S.* Typhimurium NYS lineages is described in detail below.

### A multidrug-resistant *S. *Typhimurium DT104-like lineage emerged in New York State circa 1960

One multidrug-resistant lineage possessed AMR gene profiles predictive of phenotypic resistance patterns resembling that of *S. *Typhimurium DT104 (Lineage III, n = 14); all isolates in this clade possessed AMR genes that have been shown to confer resistance to aminoglycosides, betalactams, phenicols, sulfonamides, and tetracyclines (Fig. 1). When compared to publicly available *S.* Typhimurium genomes with known phage types, all Lineage III isolates clustered among isolates belonging to *S.* Typhimurium DT104 (Supplementary Figure S5). Isolated from human and bovine hosts, this largely MDR DT104-like lineage is estimated to have emerged in NYS circa 1960 (1960.86, node height 95% HPD [1927.97, 1983.91]; Fig. 1).

Three biological processes were found to be over-represented among DT104-like Lineage III isolates relative to members of the other three lineages: “pigmentation” (GO:0043473), “conjugation” (GO:0000746), and “DNA synthesis involved in double-strand break repair via homologous recombination” (GO:0043150) (Table 1 and Supplementary Table S2). A single orthologous cluster, annotated as inner membrane protein YbiR, was associated with the biological process annotated as “pigmentation” and was exclusive to all Lineage III isolates. When compared to NCBI’s non-redundant protein sequences (nr) database using protein BLAST (BLASTP), the gene was highly similar to proteins annotated as *Salmonella* anion and citrate transporters, as well as amino acid sequences annotated as YbiR and/or a membrane protein. The over-representation of terms associated with conjugation and homologous recombination may be explained by the fact that IncFIB(S) and IncFII(S) plasmid replicons were detected in all isolates in this lineage (Fig. 1).

**Table 1:** Biological processes over-represented among four NYS *S.* Typhimurium lineages.

Lineage^a^	GO ID^b^	GO name	BH-adjusted *P*-value^c^
I	GO:0042710	Biofilm formation	5.2E−13
I	GO:0048870	Cell motility	1.1E−03
I	GO:0090101	Negative regulation of transmembrane receptor protein serine/threonine kinase signaling pathway	9.6E−03
II	GO:0009753	Response to jasmonic acid	3.9E−02
II	GO:0000746	Conjugation	3.9E−02
III	GO:0043473	Pigmentation	3.4E−07
III	GO:0000746	Conjugation	3.7E−07
III	GO:0043150	DNA synthesis involved in double-strand break repair via homologous recombination	4.0E−05
IV	GO:0019069	Viral capsid assembly	1.2E−26
IV	GO:0015694	Mercury ion transport	2.5E−10
IV	GO:0080167	Response to karrikin	6.5E−10
IV	GO:0044660	Cytolysis by virus via pore formation in host cell membrane	6.6E−10
IV	GO:0015074	DNA integration	4.1E−09
IV	GO:0019271	Aerobactin transport	1.3E−08
IV	GO:0044406	Adhesion of symbiont to host	4.7E−06
IV	GO:0009214	Cyclic nucleotide catabolic process	1.6E−04
IV	GO:0016998	Cell wall macromolecule catabolic process	2.5E−02
IV	GO:0006313	Transposition, DNA-mediated	2.6E−02

For a complete list of significant gene ontology (GO) terms in each lineage, including those referring to cellular components and molecular functions, as well as significant parent terms, see Supplementary Table S2.

^a^Lineages I (n = 37), II (n = 13), III (n = 14), and IV (n = 16).

^b^Gene Ontology (GO) identifier (ID).

^c^*P-*values obtained using Ontologizer version 2.1 and adjusted to control for the false discovery rate (FDR) at the 0.05 level using the Benjamini–Hochberg procedure.

**Figure 4:**
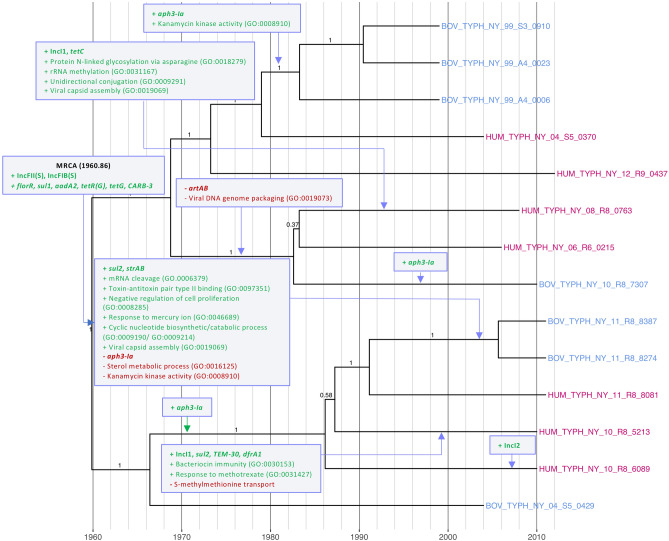
Most parsimonious acquisition and loss of selected genomic elements and predicted biological processes, superimposed on a maximum clade credibility phylogeny of 14 Lineage III (DT104-like) *S.* Typhimurium isolates from New York State. A gain or loss of a genomic element or biological process is indicated by a purple box with green or red text, respectively, with gene names and plasmid replicons in boldfaced text. Strains were isolated from bovine or human clinical sources (blue and magenta tip labels, respectively) from 1999 to 2012. The time scale in years is plotted along the x-axis of the phylogeny. Branch labels correspond to posterior probabilities.

While the pan-genome of DT104-like Lineage III differed from that of the other three major clades, there was heterogeneity within the clade as well. In addition to possessing an AMR profile characteristic of *S.* Typhimurium DT104, Lineage III was the sole lineage in which *artAB,* which encodes pertussis-like toxin ArtAB^16^, was detected. Only three Lineage III isolates (two from humans and one from a bovine host), which formed a well-supported clade within Lineage III (posterior probability = 1), did not possess genes encoding this toxin (Fig. 4). Additionally, the three Lineage III isolates in which *artAB* was not detected were the only Lineage III isolates that were not assigned the GO term representing viral DNA genome packaging (GO:0019073; Fig. 4), which is congruent with past observations that *artAB* is encoded by a prophage^17,18^. Assuming the most parsimonious explanation for its loss*, artAB* was lost in this sub-lineage sometime after 1969 (1969.74, node height 95% HPD [1944.98, 1989.25]; Fig. 4).

In addition to the presence of IncFIB(S) and IncFII(S) replicons in all Lineage III isolates, three human clinical isolates also possessed IncI replicons (Figs. 1 and 4). Assuming a most parsimonious approach to plasmid acquisition, these plasmids were acquired in their respective lineages after 1983 (Fig. 4). Two bovine isolates possessed additional resistance genes *sul2* and *strAB* (Figs. 1 and 4); these genes have been shown to confer resistance to sulfonamides and streptomycin, respectively, and often travel as a cassette^19^. Based on a parsimonious approach, they are predicted to have been acquired sometime after 1992 (1992.13, node height 95% HPD [1977.20, 2003.49]; Fig. 4).

### A multidrug-resistant *S.* Typhimurium lineage which emerged in New York State circa 1972 showcases sporadic acquisition of cephalosporin resistance-conferring genes

A little over a decade after the emergence of DT104-like Lineage III in NYS, a second lineage consisting largely of MDR isolates (Lineage IV, n = 16; Fig. 1) was predicted to have emerged (1972.39, node height 95% HPD [1948.63, 1990.43]; Fig. 1). AMR genes predictive of phenotypic resistance to aminoglycosides, betalactams, sulfonamides, and tetracyclines were present among all but one isolate in this lineage (Fig. 1).

Numerous GO terms were over-represented among the Lineage IV isolates relative to isolates from the three other major lineages (Table 1 and Supplementary Table S2). The vast majority of over-represented terms were related to viral processes, viral machinery, and the maintenance of a symbiont (Table 1 and Supplementary Table S2). A prophage most closely resembling *Escherichia* virus 186 (NCBI RefSeq Accession GCF_001500715.1) was found to be exclusive to Lineage IV (i.e., detected in all Lineage IV isolates and no other NYS *S.* Typhimurium isolates in this study; Fig. 1). Additional notable over-represented terms among Lineage IV included those related to mercury detoxification and transport, aerobactin transport, aminoglycoside phosphotransferase activity, and lysozyme activity (Table 1 and Supplementary Table S2).

**Figure 5:**
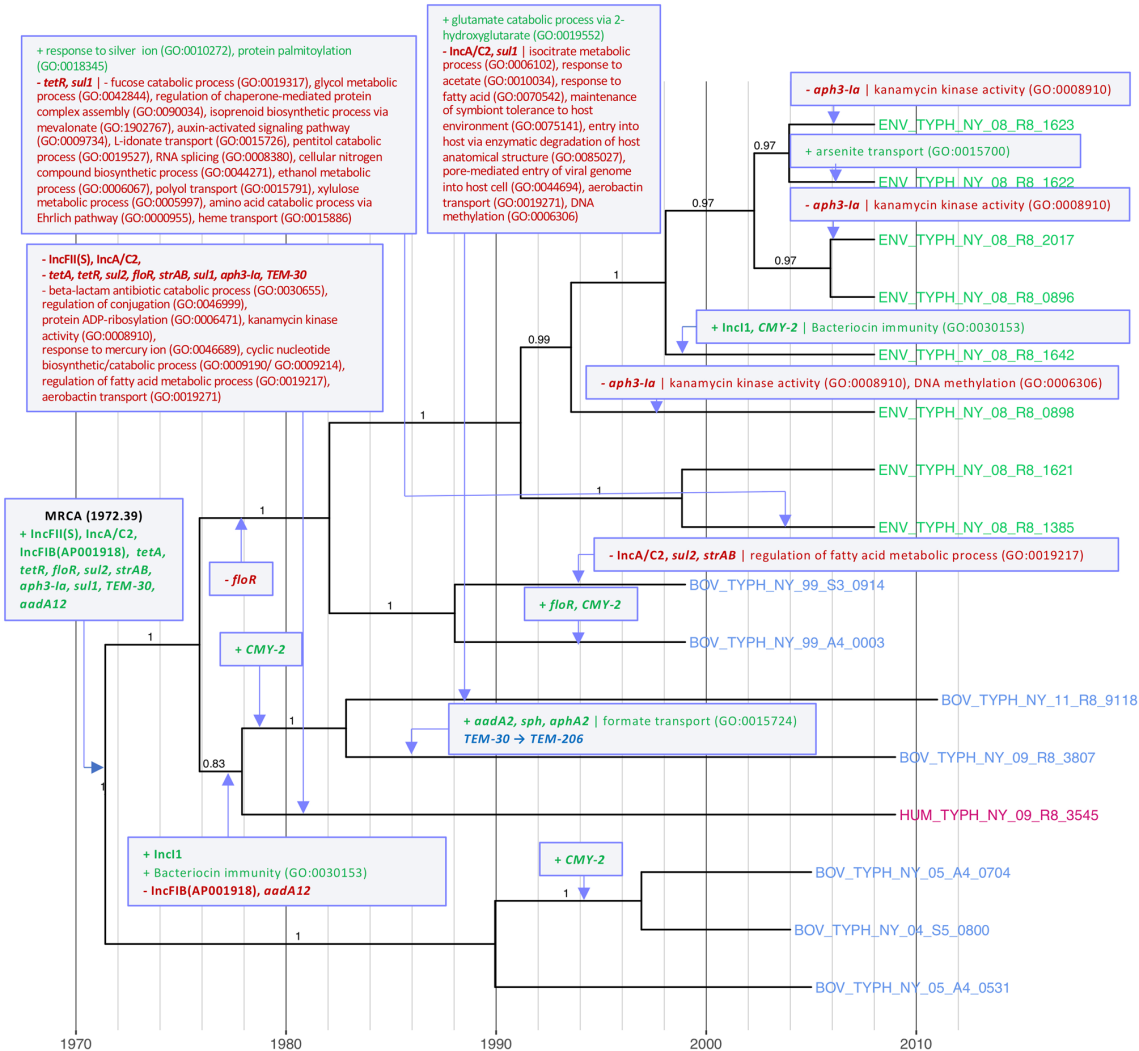
Most parsimonious acquisition and loss of selected genomic elements and predicted biological processes, superimposed on a maximum clade credibility phylogeny of 16 Lineage IV *S.* Typhimurium isolates from New York State. A gain or loss of a genomic element or biological process is indicated by a purple box with green or red text, respectively, with gene names and plasmid replicons in boldfaced text. An allele change is indicated by a box with blue text. Strains were isolated from bovine, farm environmental, or human clinical sources (blue, green, and magenta tip labels, respectively) from 1999 to 2011. The time scale in years is plotted along the x-axis of the phylogeny. Branch labels correspond to posterior probabilities.

The lone human clinical isolate in Lineage IV did not possess IncFII(S) or IncA/C2 plasmid replicons, both of which are predicted to have been lost sometime after 1978 (1978.90, node height 95% HPD [1960.48, 1994.60]; Fig. 5). Two sub-lineages, each represented by a single bovine isolate, additionally lost the IncA/C2 plasmid: the BOV_TYPH_NY_11_R8_9118 sub-lineage, which lost the plasmid, as well as the *sul1* sulfonamide resistance gene after 1983 (1983.86, node height 95% HPD [1965.538, 1997.43]; Fig. 5), and the BOV_TYPH_NY_99_S3_0914 sub-lineage, which also lost *sul2* and *strAB*, after 1989 (1989.03, node height 95% HPD [1977.65, 1997.01]; Fig. 5).

*CMY-2*, which contributes to cephalosporin resistance, was acquired by six isolates in Lineage IV (five bovine and one farm environmental isolate; Fig. 5). All *CM*Y*-2* acquisition events are predicted to have occurred in this lineage no earlier than 1978, (for BOV_TYPH_NY_11_R8_9118 and BOV_TYPH_NY_09_R8_3807; Fig. 5), while the most recent occurred after 1999 in a sub-lineage represented by a farm environmental isolate (ENV_TYPH_NY_08_R8_1642 in 1999.07, node height 95% HPD [1991.40, 2004.77]; Fig. 5).

### Sporadic introduction and loss of cephalosporin resistance-conferring *CMY-2* in a largely pan-susceptible *S.* Typhimurium lineage occurred in the 2000s

A largely pan-susceptible clade composed of a mixture of human clinical, bovine, and farm environmental isolates was detected among the 87 NYS *S.* Typhimurium isolates sequenced in this study (Lineage I, n = 37; Fig. 1). All 37 Lineage I isolates were predicted to have diverged from a common ancestor circa 1914 (1914.76, node height 95% HPD [1848.84, 1967.75]), and plasmid replicons and AMR-conferring genes were detected in this lineage only sporadically (Fig. 1). GO terms that were over-represented among Lineage I isolates relative to the other three major lineages included those relevant to biofilm formation (GO:0042710), as well as cell motility (GO:0048870) (Table 1 and Supplementary Table S2).

**Figure 6:**
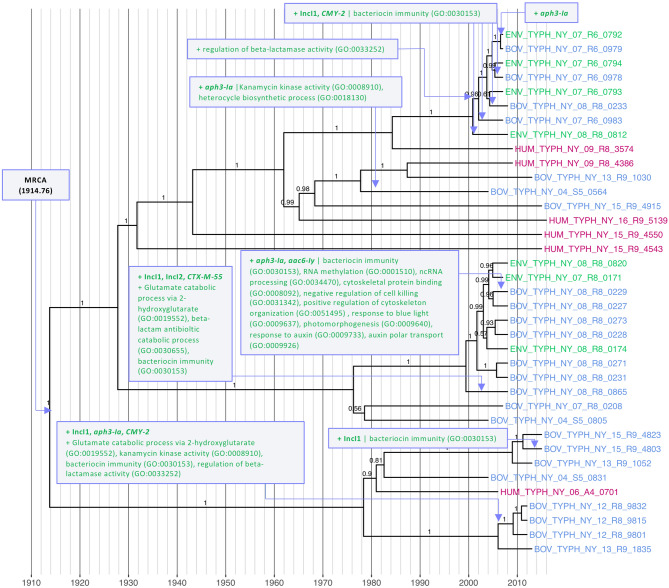
Most parsimonious acquisition and loss of selected genomic elements and predicted biological processes, superimposed on a maximum clade credibility phylogeny of 37 Lineage I *S.* Typhimurium isolates from New York State. A gain or loss of a genomic element or biological process is indicated by a purple box with green or red text, respectively, with gene names and plasmid replicons in boldfaced text. Strains were isolated from bovine, farm environmental, or human clinical sources (blue, green, and magenta tip labels, respectively) from 1999 to 2016. The time scale in years is plotted along the x-axis of the phylogeny. Branch labels correspond to posterior probabilities.

While the majority of Lineage I *S.* Typhimurium isolates displayed AMR profiles characteristic of pan-susceptible isolates, AMR genes appeared sporadically in several sub-lineages (Fig. 1). One sub-lineage, represented by bovine isolate BOV_TYPH_NY_08_R8_0865, was found to possess the *CTX-M-55* gene, which confers resistance to cephalosporins, as well as IncI1 and IncI2 replicons (Fig. 6). Using the most parsimonious explanation for its acquisition, *CTX-M-55* was acquired, along with the IncI1 and IncI2 plasmids, after 2000 (2000.43, node height 95% HPD [1993.32, 2005.43]; Fig. 6).

*CMY-2*, which also confers resistance to cephalosporins, was detected in several isolates from bovine and farm environmental sources (Fig. 1). Assuming a most parsimonious approach, one sub-lineage of bovine isolates inherited *CMY-2* on an IncI1 plasmid sometime after 2007 (2007.05, node height 95% HPD [2002.18, 2010.96]; Fig. 6). The other Lineage I sub-lineage in which *CMY-2* was detected was a clade of four bovine and four farm environmental isolates (8 total isolates), which displayed a mixture of isolates with and without the gene, also on IncI1 plasmids (Fig. 1). Although there were multiple parsimonious explanations for *CMY-2* inheritance and loss in this clade that would have led to its possession by three bovine and one environmental isolate, all involve inheritance after 1985 (1985.28, node height 95% HPD [1967.45, 1999.86]; Fig. 6).

### A mixed susceptible/MDR *S.* Typhimurium lineage was introduced into the NYS bovine population circa 1960

A well-supported lineage of 13 *S.* Typhimurium isolates from bovine and human clinical sources (Lineage II, n = 13; Fig. 1) was predicted to have emerged in NYS in 1882 (1882.00, node height 95% HPD [1789.40, 1950.77]). IncFIB(S) and IncFII(S) plasmid replicons were detected in 11 of the 13 isolates in this clade, while the appearance of additional plasmid replicons and AMR determinants within the lineage occurred sporadically (Fig. 1). GO terms over-represented among isolates in this lineage relative to all other isolates were “response to jasmonic acid” (GO:0009753), and “conjugation” (GO:0000746) (Table 1 and Supplementary Table S2).

**Figure 7:**
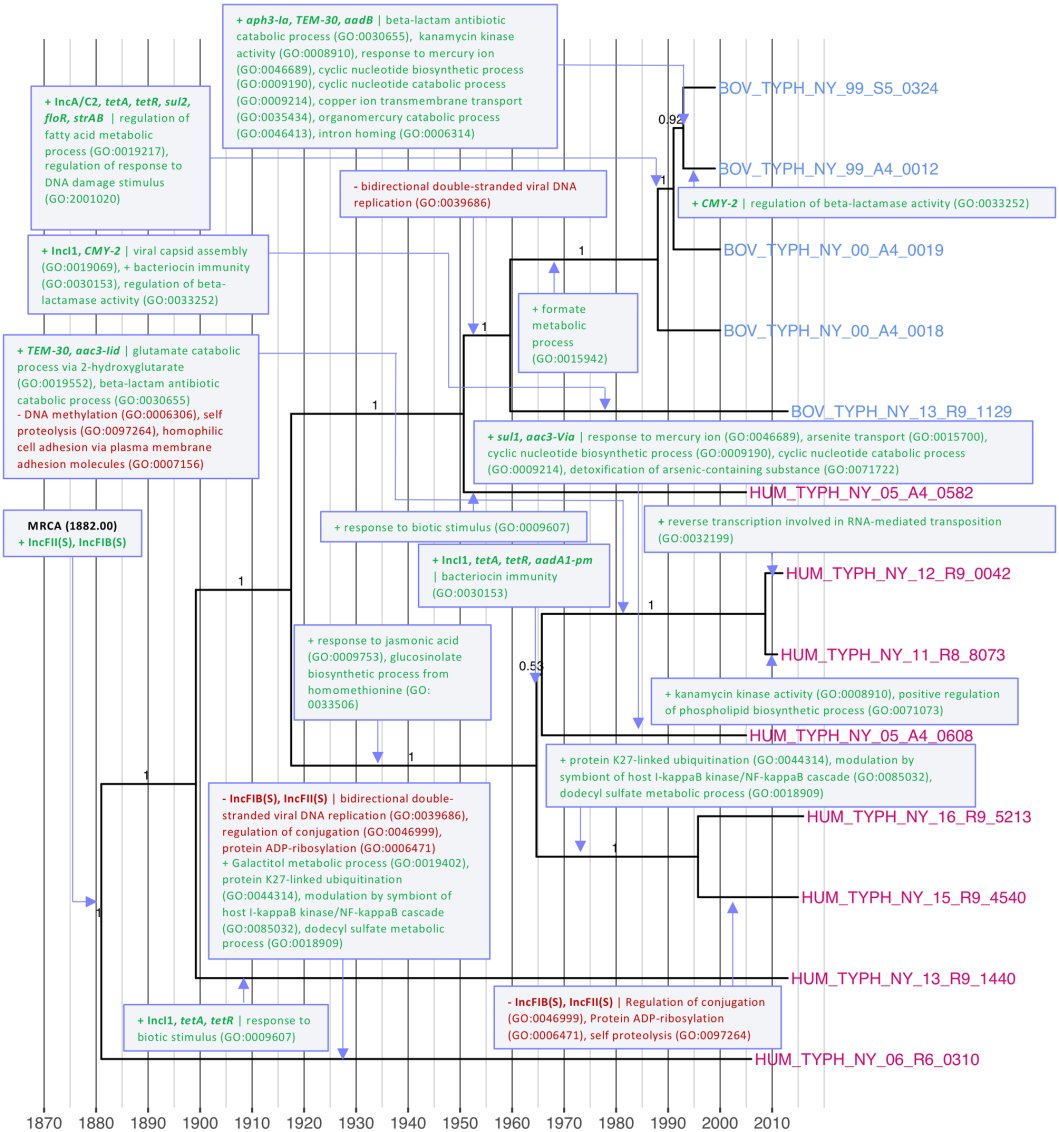
Most parsimonious acquisition and loss of selected genomic elements and predicted biological processes, superimposed on a maximum clade credibility phylogeny of 13 Lineage II *S.* Typhimurium isolates from New York State. A gain or loss of a genomic element or biological process is indicated by a purple box with green or red text, respectively, with gene names and plasmid replicons in boldfaced text. Strains were isolated from bovine or human clinical sources (blue and magenta tip labels, respectively) from 1999 to 2016. The time scale in years is plotted along the x-axis of the phylogeny. Branch labels correspond to posterior probabilities.

Lineage II appears to have been first introduced into the NYS bovine population circa 1960 (1960.63, node height 95% HPD [1926.09, 1986.76]), after which AMR determinants were introduced into the population on numerous separate occasions (Fig. 7). Three bovine sub-lineages acquired an IncA/C2 plasmid, along with AMR genes that confer resistance to tetracyclines, sulfonamides, phenicols, and streptomycin (*tetAR*, *sul2*, *floR,* and *strAB*, respectively), after 1992 (1992.04, node height 95% HPD [1985.18, 1997.12]; Fig. 7). Two of the three bovine isolates acquired additional genes conferring resistance to beta-lactams and aminoglycosides (*TEM-30*,* aph3-Ia*, and *aadB*) within the same time frame (Fig. 7). One of these two bovine isolates, BOV_TYPH_NY_99_A4_0012, additionally carried *CMY-2*, which is predicted to have been acquired after 1993 (1993.93, node height 95% HPD [1988.48, 1998.13]; Fig. 7). *CMY-2* and an IncI1 replicon were additionally detected in bovine isolate BOV_TYPH_NY_13_R9_1129, and they are predicted to have been acquired sometime after Lineage II’s introduction in the bovine population circa 1960 (Fig. 7).

The acquisition of AMR determinants was not limited to bovine sub-lineages, as human clinical sub-lineages of Lineage II showcased sporadic acquisition of AMR determinants as well. IncI1 replicons and *tetAR* appeared in human sub-lineages on two separate occasions: once in the sub-lineage represented by HUM_TYPH_NY_13_R9_1440, sometime after 1900 (1900.19, node height 95% HPD [1819.10, 1957.74]; Fig. 7), and again, alongside an additional aminoglycoside resistance gene (*aadA1-pm*), in a sub-lineage of three human isolates sometime after 1966 (1966.72, node height 95% HPD [1933.86, 1994.57]; Fig. 7). Two of the three human isolates in the latter group additionally possessed beta-lactamase *TEM-30* and aminoglycoside resistance gene *aac3-Iid*, while the third human isolate (HUM_TYPH_NY_05_A4_0608) acquired sulfonamide and aminoglycoside resistance genes *sul1* and *aac3-*Via, respectively, close to within the same time frame (Fig. 7).

## Discussion

In terms of the population structure of human- and bovine-associated *S.* Typhimurium, the genomic diversity encompassed by the 87 NYS isolates queried in this study mirrored the diversity observed in human- and bovine-associated *S.* Typhimurium from the U.S. as a whole: all six major U.S. *S.* Typhimurium lineages were represented among the 87 NYS *S.* Typhimurium strains characterized here. Furthermore, the majority of the NYS isolates could be placed into one of four major *S.* Typhimurium lineages which clustered by AMR profile, as well as the pan genome as a whole. These results are consistent with historical observations of the population structure of NYS *S.* Typhimurium from livestock (primarily cattle), as four *S.* Typhimurium clonal groups (i.e., phage types) were predominant in NYS from 1973 to 1981^20^.

In this study, two MDR *S.* Typhimurium lineages are predicted to have emerged in NYS in the latter half of the twentieth century, one of which was DT104-like Lineage III. Lineage III was predicted to have emerged in New York State circa 1960, which falls within the timeframe of emergence for DT104 proposed by others. Leekitcharoenphon et al.^12^ estimated that DT104 emerged globally as antimicrobial susceptible circa 1948, later becoming MDR DT104 circa 1972, while Mather et al.^14^ estimated that DT104 from Scotland shared a most recent common ancestor that dates to 1968. All of these estimates pre-date the global MDR *S.* Typhimurium DT104 epidemic that occurred in the 1990s^13,21^, suggesting that it took at least a decade for the MDR version of the pathogen to begin to appear and/or be detected in human clinical settings. In the case of MDR Lineage IV, which emerged in NYS circa 1972, it is possible that the lineage first emerged as antimicrobial susceptible and later acquired AMR genes, as was the case for *S.* Typhimurium DT104. Future studies querying a larger sample of *S.* Typhimurium Lineage IV strains isolated over a longer time frame will offer further insight into the evolution of this lineage, as well as the temporal acquisition of AMR.

In addition to the emergence of two MDR *S.* Typhimurium lineages in NYS in the twentieth century, two largely AMR-susceptible lineages were also identified, both of which are predicted to have emerged prior to their MDR counterparts. Lineage I and II emerged in NYS circa 1914 and 1882, respectively, coinciding with the rapid industrialization of the dairy industry in the state^22^. Fluid milk, which few farmers had commodified in NYS prior to the mid-nineteenth century, was regularly shipped to New York City (NYC) via railroad beginning in the 1840s^22^. As railroad services expanded after 1850, farmers transitioned from subsistence farming to running large-scale, specialized dairy operations, delegating practices such as cheese production to an increasing number of factories throughout the state^22^. In 1884, roughly coinciding with the time at which Lineage II is predicted to have emerged, NYS began to regulate the growing dairy industry, with the formation of what would eventually be known as the Department of Agriculture that year^22^. Notable early regulations included permit requirements for milk dealers (1896), regular inspection of farms and processing plants (1906), and mandatory pasteurization (1911)^22^, all of which occurred shortly prior to the predicted time of emergence of Lineage I in the state. The results presented here indicate that industrialization, urbanization, and increased movement between city, farm, and factory in New York State may have contributed to the spread of *S.* Typhimurium in NYS, similar to a phenomenon observed in *Salmonella enterica* serotype Enteritidis^23^.

AMR determinants were acquired within the largely antimicrobial-susceptible Lineages I and II on numerous occasions. In both Lineage I and II, the introduction of the *CMY-2* gene in multiple sub-lineages within the second half of the twentieth century, as well as after 2000, was observed. This coincides with a worldwide increase in infections caused by extended spectrum β-lactamase (ESBL) and AmpC β-lactamase–producing bacteria^24,25^, as well as an increase in isolation of these organisms from foods of animal origin^24,26^. *CMY-2,* a plasmid-encoded AmpC beta-lactamase, can confer resistance to cephalosporins, including ceftriaxone and ceftiofur^24,27,28^. Ceftriaxone is used to treat invasive salmonellosis in humans, including children, if fluoroquinolones cannot be used^29^. Previously marketed as Rocephin, ceftriaxone was discovered in the late 1970s and has been used in human medicine since the early 1980s^30–32^, after a worldwide patent was filed in 1979^32^. Ceftiofur, however, is used in veterinary settings to treat diseases in animals, including dairy cattle^28,33^. First described in 1987^34^, ceftiofur first garnered FDA approval in 1988 and is available only by veterinarian prescription for FDA-approved use (e.g., clinical mastitis and respiratory disease in dairy cattle)^35,36^. There has been concern that the use of ceftiofur in farm animals can contribute to the dissemination of *CMY*-2 and, thus, bacteria that are co-resistant to ceftriaxone^27,28,37^. Our results confine the acquisition of *CMY*-2 in NYS *S.* Typhimurium largely to the era of ceftriaxone and ceftiofur usage, with nearly all events occurring after 1985 using the most parsimonious explanation for its acquisition.

Antibiotic consumption has played a significant role in the propagation of antimicrobial resistant bacteria^38,39^. Here, we used WGS to characterize a foodborne pathogen from human, livestock, and livestock-adjacent sources over an 18-year period in a confined geographical region. We observed numerous AMR acquisition events, the vast majority of which occurred in the latter half of the twentieth century, as well as into the twenty-first century. Our findings support the hypothesis that AMR gene acquisition events can occur widely in a pathogen group and indicate that emergence and dispersal of AMR *Salmonella* may involve multiple acquisition events. Future studies incorporating WGS data from additional *S.* Typhimurium isolates from this region will aid in refining and expanding upon these estimates. Furthermore, additional sequences will allow for the quantitative characterization of horizontal transmission of AMR determinants within the serotype, making it possible to predict the rate at which AMR gene acquisition and loss occurs.

## Methods

### Isolate selection

A total of 42 isolates available in Food Microbe Tracker^40^ that had been previously serotyped as *S.* Typhimurium were stratified by year and selected using random numbers generated using the sample function in R version 3.4.3^41^. Isolates came from either bovine (clinical or non-clinical) or human (clinical) sources in New York State and were isolated between 1999 and 2016. Following WGS (see “Whole-genome sequencing” below), these data were supplemented with WGS data from an additional 45 *S.* Typhimurium isolates from bovine (clinical or non-clinical), human (clinical), or bovine farm environmental sources in New York State that had been isolated within the same time frame and sequenced as described by Carroll et al.^27^, yielding WGS data from a total of 87 *S.* Typhimurium isolates from New York State (Supplementary Table S1).

### Whole-genome sequencing

The 42 isolates selected using stratified random sampling were sequenced through a partnership with the *Salmonella* Foodborne Syst-OMICS consortium^42^. Isolates were submitted to the Plateforme d’Analyses Génomiques of the Institut de Biologie Intégrative et des Systèmes (Université Laval, Quebec, Canada), where they underwent paired-end sequencing (300 bp reads) using an Illumina MiSeq platform as described by Emond-Rheault et al.^42^.

### Initial data processing and genome assembly

Illumina sequencing adapters and low-quality bases were trimmed using Trimmomatic version 0.33 for TruSeq paired-end reads^43^. FastQC version 0.11.5 (https://www.bioinformatics.babraham.ac.uk/projects/fastqc/) was used to confirm that all adapter sequences had been removed, and genomes were assembled de novo using SPAdes version 3.8.0^44^. Average per-base assembly coverage was determined by mapping reads back to their respective assemblies using BWA mem version 0.7.15^45,46^ and samtools version 1.5^47^.

### In silico serotyping, multi-locus sequence typing, antimicrobial resistance gene detection, plasmid replicon detection, prophage detection, and virulence factor detection

In silico serotyping and core genome multi-locus sequence typing (cgMLST) were performed using the SISTR version 1.0.2 command line tool^48^, while 7-gene in silico multi-locus sequence typing (MLST) was performed using the *Salmonella enterica* scheme available through PubMLST^49^ and nucleotide BLAST (blastn)^50^, as implemented in seq2mlst (https://github.com/lmc297/seq2mlst). Antimicrobial resistance (AMR) genes were detected in all assembled genomes using BTyper version 2.2.0^51^, which uses blastn version 2.6.0^50^ and the ARG-ANNOT database version 3^52^ and selects the allele with the highest blast bit score for a detected AMR gene. A gene was considered to be present in a genome if it was detected at ≥ 75% and 50% identity and coverage, respectively, as these cutoffs have been shown to correlate highly with phenotypic resistance of *S.* Typhimurium to 12 antimicrobials^27^. AMR-conferring chromosomal point mutations in *S. enterica* that have been described in Zankari et al*.*^53^ were also queried using Roary version 3.11.0^54^, and the resulting gene sequences were visualized in MEGA7^55^. To detect plasmid replicons in all genomes, replicons in the PlasmidFinder Enterobacteriaceae database (last updated 14 February 2018)^56^ were used in conjunction with the nucleotide blast^50^ function as implemented in BTyper version 2.2.0^51^. A replicon was considered to be present in a genome if matched with at least 80% and 60% identity and coverage, respectively. Prophage were detected by submitting each genome assembly to the PHASTER web server^57^ via the URLAPI. Only “intact” prophage were considered to be present (i.e., prophage hits classified as “questionable” or “incomplete” were excluded). Virulence factors were detected in each genome using ABRicate version 0.8 (https://github.com/tseemann/abricate) and the Virulence Factors Database (VFDB) (June 11, 2018)^58^, using minimum identity and coverage thresholds of 80 and 50%, respectively.

### Variant calling and filtering

The closed chromosome of NCBI reference genome *S.* Typhimurium strain LT2 (NCBI RefSeq assembly accession GCF_000006945.2) was used as a reference genome for variant calling. FastANI version 1.0^59^ was used to calculate average nucleotide identity (ANI) values between each of the *S.* Typhimurium genomes in this study relative to the *S.* Typhimurium str. LT2 reference chromosome, with the resulting ANI values ranging from 99.87 to 99.98. Trimmed Illumina paired-end reads were mapped to the *S.* Typhimurium str. LT2 reference chromosome using BWA mem version 0.7.15^45,46^, and variants were called using samtools version 1.5^47^ and bcftools version 1.3.1^60^. Vcftools version 0.1.14^61^ was used to remove indels and low-quality SNPs (i.e., a Phred quality score < 20) from the data set and to construct consensus sequences. Gubbins version 2.2.0^62^ was used to filter out recombination events from the consensus sequences, yielding a total of 5,254 SNPs among the 87 *S.* Typhimurium isolates, 4,774 of which were core SNPs. The variant calling pipeline described here has been implemented as the default pipeline in SNPBac version 1.0.0 (https://github.com/lmc297/SNPBac )^63^.

### Initial phylogeny construction and temporal diagnostics

IQ-TREE version 1.6.5^64^ was used to construct a maximum likelihood (ML) phylogeny using (1) the set of filtered SNPs produced by Gubbins version 2.2.0 for all 87 genomes, (2) the optimal ascertainment bias-aware nucleotide substitution model determined using Bayesian information criteria (BIC) values produced with ModelFinder (the TVMe + ASC + R4 model)^65^, (3) 1,000 replicates of the Shimodaira–Hasegawa–like approximate likelihood ratio test (SH-aLRT) of branch support^66^, and (4) 1,000 replicates of the ultrafast bootstrap test^67^. TempEst version 1.5^68^ was used to assess the temporal structure of the resulting phylogeny. Due to the lack of evidence of a strong temporal signal for the phylogeny constructed using all 87 isolates (*R*^2^ < 0.1), a relaxed molecular clock was used to account for varying rates on branches (see “Temporal phylogeny construction” section below).

### Temporal phylogeny construction

BEAST version 2.5.0^69^ was used to construct a tip-dated phylogeny using SNPs detected in the 87 NYS *S.* Typhimurium isolate genomes, using an initial clock rate of 2.79 × 10^−7^ substitutions/site/year^12^ and an ascertainment bias correction to account for the use of solely variant sites (https://groups.google.com/forum/#!topic/beast-users/QfBHMOqImFE). bmodeltest^70^ was used to explore the transition-transversion model space and infer an average substitution model. A relaxed lognormal molecular clock^71^ and a Bayesian skyline population model^72^ were used to account for varying rates between lineages and potential changes in effective population size, respectively. A log-normal distribution with a mean of 8.6 × 10^−7^ and standard deviation of 1.5 (median of 2.79 × 10^−7^) was used as the prior on the uncorrelated log-normal relaxed molecular clock mean rate parameter (ucld.mean). Five independent runs using this clock/population model combination were performed, using chain lengths of 1 billion generations, sampling every 1 million generations. LogCombiner-2^69^ was used to aggregate the resulting log and tree files produced, and TreeAnnotator-2^69^ was used to produce a maximum clade credibility (MCC) tree using Common Ancestor node heights and 10% burn-in. The phylogeny was annotated using R version 3.5.0^41^ and the following packages: ggplot2^73^, ggtree^74^, phylobase^75^, and treeio^76^. The BEAST2 XML file, combined log file, and annotated tree file are deposited at https://github.com/lmc297/NYS_Typhimurium_2018. A maximum parsimony approach was used to predict gain and loss of AMR and other genomic determinants, as well as predicted functions, using the resulting time-scaled MCC phylogeny.

### Genome annotation and functional characterization

All genomes were annotated using Prokka version 1.12^77^, and Roary version 3.11.0^54^ was used to identify orthologous clusters of genes and produce an orthologous cluster presence/absence matrix. The resulting orthologous clusters were functionally annotated using blast2go version 1.2.1^78^ and the Uniprot database^79^. For protein sequences that could not be assigned using Uniprot, the RefSeq protein database^80^ was used in conjunction with blast2go. Ontologizer version 2.1^81^ was used to determine whether any GO terms were overrepresented among isolates representing each of the four, well-supported *S.* Typhimurium lineages, with the Parent–Child Union method^82^ used to assess enrichment of terms, and a Benjamini–Hochberg correction to control the false discovery rate (FDR)^83^. Non-metric multidimensional scaling (NMDS)^84,85^ was used to collapse the orthologue and GO-term presence/absence matrices into 2 dimensional space using besPLOT (https://github.com/lmc297/besPLOT), a Jaccard distance metric, and the following packages in R version 3.4.3: ggplot2^73^, shiny^86^, vegan^87^, plyr^88^, dplyr^89^, cluster^90^, and ggrepel^91^.

### Additional statistical analyses

All additional statistical analyses were conducted using R version 3.5.0^41^. To assess the validity of each isolate’s assignment into one of four well-supported lineages based on AMR presence/absence profile, rhierbaps^92,93^ was used to assign each of the 87 isolates to a cluster using SNPs identified in the 87 *S.* Typhimurium genomes (see “Variant calling and filtering” section above). The top-level cluster assignments determined using rhierbaps were in agreement with isolate assignment into the four major AMR profile-based lineages for all but one isolate (isolate HUM_TYPH_NY_10_R8_5386 was assigned to Lineage II using rhierbaps but was excluded from the lineage based on the topology of the phylogeny; Fig. 1).

Analysis of similarity (ANOSIM)^94^ and PERMANOVA^95^ were used to assess the clustering of isolates based on presence/absence profiles of (1) orthologous clusters assigned using Roary, and (2) gene ontology (GO) terms assigned using blast2go. The betadisper and anova functions in R’s vegan and stats packages, respectively, were used to ensure that the dispersions for one or more of the four lineages was not different^96^. ANOSIM was used to test whether the average ranks of within-lineage distances for the four selected, well-supported lineages in the *S.* Typhimurium phylogeny that appeared to differ by AMR profile (Fig. 1) were greater than or equal to the average ranks of between-lineage distances^97^. Three independent ANOSIM runs using the anosim function in R’s vegan^87^ package were performed, each using 10,000 permutations with Jaccard dissimilarities. Pairwise ANOSIM and betadisper/anova tests were conducted following a significant test statistic, with a Bonferroni correction used to correct for multiple comparisons. PERMANOVA was performed to test whether the centroids of the four selected lineages were equivalent for all groups^97^ based on presence/absence of (1) orthologous gene clusters and (2) GO terms, using the adonis function in R's vegan package and three independent runs of 10,000 permutations with Jaccard dissimilarities. Pairwise PERMANOVA and betadisper/anova tests were conducted following a significant test statistic, with a Bonferroni correction used to correct for multiple comparisons.

### Phylogenomic comparison of NYS *S.* Typhimurium to publicly available human- and bovine-associated *S.* Typhimurium from the United States

To determine where bovine- and human-associated NYS *S.* Typhimurium fell within the topology of bovine- and human-associated *S.* Typhimurium from the United States as a whole, all genome assemblies meeting the following criteria were downloaded via Enterobase^98^ (accessed November 29, 2018): (1) genomes belonged to the *S.* Typhimurium serotype, as determined in silico using the implementation of SISTR^48^ in Enterobase^98^, (2) the country of isolation was the United States, and (3) the isolation source was either “Human” or “Bovine”, as recorded in Enterobase’s “Source Niche” and “Source Type” fields, respectively. This produced an additional 1,123 assembled genomes (664 and 459 from bovine- and human-associated sources, respectively). Four outlier genomes (three from humans and one bovine-associated) that were downloaded via Enterobase were excluded from further analysis, yielding a total of 1,206 genomes. Parsnp version 1.2^99^ was used to identify core SNPs among the 1,207 U.S. bovine- and human-associated *S.* Typhimurium genomes (1,119 genomes from Enterobase, 87 NYS *S.* Typhimurium genomes associated with this project, and the *S.* Typhimurium strain LT2 chromosome, which was used as a reference), and Parsnp’s implementation of PhiPack^100^ was used to filter out recombination. A ML phylogeny was constructed using (1) the resulting core SNPs (n = 18,493) and IQ-TREE version 1.6.5^64^, (2) the optimal nucleotide substitution model determined using BIC values produced with ModelFinder (the TVM + F + ASC + R2 model)^65^, (3) 1,000 SH-aLRT replicates^66^, and (4) 1,000 ultrafast bootstrap replicates^67^. Additionally, rhierbaps^92,93^ was used to assign each of 1,207 genomes to a cluster using the core SNPs identified by Parsnp.

### Phylogenomic comparison of NYS *S.* Typhimurium to publicly available *S.* Typhimurium genomes with assigned phage types

All genomes with a listed phage type that had been serotyped as *S.* Typhimurium using the implementation of SISTR available in Enterobase were downloaded (accessed February 10, 2019; n = 322). Nine outlier genomes were excluded, leaving a total of 313 publicly available genomes with listed phage types. Parsnp version 1.2 was used to identify core SNPs among the 401 *S.* Typhimurium genomes (313 genomes from Enterobase, 87 NYS *S.* Typhimurium genomes associated with this project, and the *S.* Typhimurium strain LT2 chromosome, which was used as a reference), and Parsnp’s implementation of PhiPack was used to filter out recombination. A ML phylogeny was constructed using (1) the resulting core SNPs (n = 15,919) and IQ-TREE version 1.6.5, (2) the optimal nucleotide substitution model determined using BIC values produced with ModelFinder (the TVM + F + ASC + R2model), (3) 1,000 SH-aLRT replicates, and (4) 1,000 ultrafast bootstrap replicates. The resulting phylogeny was displayed and annotated using FigTree version 1.4.3 (https://tree.bio.ed.ac.uk/software/figtree/).

## Supplementary information

Supplementary Information.

## Data Availability

Illumina reads for all *S.* Typhimurium isolates used in this study are available in the National Center for Biotechnology Information (NCBI) Sequence Read Archive (SRA)^101^ under Bioproject accession numbers PRJNA497340 and PRJNA308370. The XML file used to conduct all analyses in BEAST, as well as the resulting combined log file and annotated tree file, are deposited at https://github.com/lmc297/NYS_Typhimurium_2018.

## References

[1] CDC. National *Salmonella* Surveillance Annual Report, 2016 (Atlanta, Georgia, 2018).

[2] Rabsch W (2002). *Salmonella enterica* serotype Typhimurium and its host-adapted variants. Infect. Immun..

[3] Mueller-Doblies D, Speed KCR, Kidd S, Davies RH (2018). *Salmonella* typhimurium in livestock in Great Britain—trends observed over a 32-year period. Epidemiol. Infect..

[4] Suar M (2006). Virulence of broad- and narrow-host-range *Salmonella enterica* serovars in the streptomycin-pretreated mouse model. Infect. Immun..

[5] Baker S, Thomson N, Weill FX, Holt KE (2018). Genomic insights into the emergence and spread of antimicrobial-resistant bacterial pathogens. Science.

[6] Rabsch W (2007). *Salmonella* typhimurium phage typing for pathogens. Methods Mol. Biol..

[7] Andrews-Polymenis HL (2004). Host restriction of *Salmonella enterica* serotype Typhimurium pigeon isolates does not correlate with loss of discrete genes. J. Bacteriol..

[8] Pasmans F (2004). Assessment of virulence of pigeon isolates of *Salmonella enterica* subsp. enterica serovar typhimurium variant copenhagen for humans. J. Clin. Microbiol..

[9] Boonyarittichaikij R (2017). *Salmonella* Typhimurium DT193 and DT99 are present in great and blue tits in Flanders Belgium. PLoS ONE.

[10] Farzan A (2008). Molecular epidemiology and antimicrobial resistance of *Salmonella* typhimurium DTI04 on Ontario swine farms. Can. J. Vet. Res..

[11] Bergeron N, Corriveau J, Letellier A, Daigle F, Quessy S (2010). Characterization of *Salmonella* Typhimurium isolates associated with septicemia in swine. Can. J. Vet. Res..

[12] Leekitcharoenphon P (2016). Global genomic epidemiology of *Salmonella enterica* serovar typhimurium DT104. Appl. Environ. Microbiol..

[13] Threlfall EJ (2000). Epidemic *Salmonella typhimurium* DT 104—a truly international multiresistant clone. J. Antimicrob. Chemother..

[14] Mather AE (2013). Distinguishable epidemics of multidrug-resistant *Salmonella* Typhimurium DT104 in different hosts. Science.

[15] Okoro CK (2012). Intracontinental spread of human invasive *Salmonella* Typhimurium pathovariants in sub-Saharan Africa. Nat. Genet..

[16] Saitoh M (2005). The *artAB* genes encode a putative ADP-ribosyltransferase toxin homologue associated with *Salmonella enterica* serovar Typhimurium DT104. Microbiology.

[17] Uchida I (2009). *Salmonella enterica* serotype Typhimurium DT104 ArtA-dependent modification of pertussis toxin-sensitive G proteins in the presence of [32P]NAD. Microbiology.

[18] Tamamura Y, Tanaka K, Uchida I (2017). Characterization of pertussis-like toxin from *Salmonella* spp. that catalyzes ADP-ribosylation of G proteins. Sci. Rep..

[19] Bean DC, Livermore DM, Hall LM (2009). Plasmids imparting sulfonamide resistance in *Escherichia coli*: implications for persistence. Antimicrob. Agents Chemother..

[20] McDonough PL, Timoney JF, Jacobson RH, Khakhria R (1989). Clonal groups of *Salmonella* typhimurium in New York State. J. Clin. Microbiol..

[21] Helms, M., Ethelberg, S., Molbak, K. & Group, D. T. S (2005). International *Salmonella* Typhimurium DT104 infections, 1992–2001. Emerg. Infect. Dis..

[22] Eisenstadt PR, Moss L-E (2005). The Encyclopedia of New York State.

[23] Deng X (2014). Genomic epidemiology of *Salmonella enterica* serotype Enteritidis based on population structure of prevalent lineages. Emerg. Infect. Dis..

[24] Liebana E (2013). Public health risks of enterobacterial isolates producing extended-spectrum beta-lactamases or AmpC beta-lactamases in food and food-producing animals: an EU perspective of epidemiology, analytical methods, risk factors, and control options. Clin. Infect. Dis..

[25] Pitout JD, Laupland KB (2008). Extended-spectrum beta-lactamase-producing Enterobacteriaceae: an emerging public-health concern. Lancet Infect. Dis..

[26] Bergenholtz RD, Jorgensen MS, Hansen LH, Jensen LB, Hasman H (2009). Characterization of genetic determinants of extended-spectrum cephalosporinases (ESCs) in *Escherichia coli* isolates from Danish and imported poultry meat. J. Antimicrob. Chemother..

[27] Carroll LM (2017). Whole-genome sequencing of drug-resistant *Salmonella enterica* isolates from Dairy Cattle and Humans in New York and Washington States reveals source and geographic associations. Appl. Environ. Microbiol..

[28] Alcaine SD (2005). Ceftiofur-resistant *Salmonella* strains isolated from dairy farms represent multiple widely distributed subtypes that evolved by independent horizontal gene transfer. Antimicrob. Agents Chemother..

[29] Yang WC (2016). Development of ceftriaxone resistance in *Salmonella enterica* serotype Oranienburg during therapy for bacteremia. J. Microbiol. Immunol. Infect..

[30] Landau R, Achilladelis B, Scriabine A (1999). Pharmaceutical Innovation: Revolutionizing Human Health.

[31] Roche. *Our History: Launch of Rocephin*. https://www.roche.com/about/history.htm#reform_1979. Accessed 15 Feb 2019.

[32] Mörgeli C, Päuser S, Mörgeli C, Schaad UB (2012). Lifesavers for Millions.

[33] Hornish RE, Kotarski SF (2002). Cephalosporins in veterinary medicine - ceftiofur use in food animals. Curr. Top. Med. Chem..

[34] Yancey RJ (1987). Ceftiofur sodium, a broad-spectrum cephalosporin: evaluation in vitro and in vivo in mice. Am. J. Vet. Res..

[35] Zoetis (2014). *The Facts About Ceftiofur: Antibiotic Stewardship and Safety*. https://www.dairywellness.com/pdfs/Ceftiofur_FactSheet_120314_FINAL.pdf. Accessed 15 Feb 2019.

[36] FDA (2018). *Extralabel Use and Antimicrobials*. https://www.fda.gov/AnimalVeterinary/SafetyHealth/AntimicrobialResistance/ucm421527.htm. Accessed 15 Feb 2019.

[37] Tragesser LA, Wittum TE, Funk JA, Winokur PL, Rajala-Schultz PJ (2006). Association between ceftiofur use and isolation of *Escherichia coli* with reduced susceptibility to ceftriaxone from fecal samples of dairy cows. Am. J. Vet. Res..

[38] Van Boeckel TP (2015). Global trends in antimicrobial use in food animals. Proc. Natl. Acad. Sci. USA.

[39] Klein EY (2018). Global increase and geographic convergence in antibiotic consumption between 2000 and 2015. Proc. Natl. Acad. Sci. USA.

[40] Vangay P, Fugett EB, Sun Q, Wiedmann M (2013). Food microbe tracker: a web-based tool for storage and comparison of food-associated microbes. J. Food Prot..

[41] R Core Team (2018). R: A Language and Environment for Statistical Computing.

[42] Emond-Rheault JG (2017). A Syst-OMICS approach to ensuring food safety and reducing the economic burden of Salmonellosis. Front. Microbiol..

[43] Bolger AM, Lohse M, Usadel B (2014). Trimmomatic: a flexible trimmer for Illumina sequence data. Bioinformatics.

[44] Bankevich A (2012). SPAdes: a new genome assembly algorithm and its applications to single-cell sequencing. J. Comput. Biol..

[45] Li, H. Aligning Sequence Reads, Clone Sequences and Assembly Contigs with BWA-MEM. arXiv:1303.3997v1[q-bio.GN] (2013).

[46] Li H, Durbin R (2010). Fast and accurate long-read alignment with Burrows-Wheeler transform. Bioinformatics.

[47] Li H (2009). The sequence alignment/map format and SAMtools. Bioinformatics.

[48] Yoshida CE (2016). The *Salmonella *in silico typing resource (SISTR): an open web-accessible tool for rapidly typing and subtyping draft *Salmonella* genome assemblies. PLoS ONE.

[49] Jolley KA, Maiden MC (2010). BIGSdb: scalable analysis of bacterial genome variation at the population level. BMC Bioinform..

[50] Camacho C (2009). BLAST+: architecture and applications. BMC Bioinform..

[51] Carroll LM, Kovac J, Miller RA, Wiedmann M (2017). Rapid, high-throughput identification of anthrax-causing and emetic *Bacillus cereus* group genome assemblies using BTyper, a computational tool for virulence-based classification of *Bacillus cereus* group isolates using nucleotide sequencing data. Appl. Environ. Microbiol..

[52] Gupta SK (2014). ARG-ANNOT, a new bioinformatic tool to discover antibiotic resistance genes in bacterial genomes. Antimicrob. Agents Chemother..

[53] Zankari E (2017). PointFinder: a novel web tool for WGS-based detection of antimicrobial resistance associated with chromosomal point mutations in bacterial pathogens. J. Antimicrob. Chemother..

[54] Page AJ (2015). Roary: rapid large-scale prokaryote pan genome analysis. Bioinformatics.

[55] Kumar S, Stecher G, Tamura K (2016). MEGA7: molecular evolutionary genetics analysis version 7.0 for bigger datasets. Mol. Biol. Evol..

[56] Carattoli A (2014). In silico detection and typing of plasmids using PlasmidFinder and plasmid multilocus sequence typing. Antimicrob. Agents Chemother..

[57] Arndt D (2016). PHASTER: a better, faster version of the PHAST phage search tool. Nucl. Acids Res..

[58] Chen L (2005). VFDB: a reference database for bacterial virulence factors. Nucl. Acids Res..

[59] Jain C, Rodriguez RL, Phillippy AM, Konstantinidis KT, Aluru S (2018). High throughput ANI analysis of 90K prokaryotic genomes reveals clear species boundaries. Nat. Commun..

[60] Li H (2011). A statistical framework for SNP calling, mutation discovery, association mapping and population genetical parameter estimation from sequencing data. Bioinformatics.

[61] Danecek P (2011). The variant call format and VCFtools. Bioinformatics.

[62] Croucher NJ (2015). Rapid phylogenetic analysis of large samples of recombinant bacterial whole genome sequences using Gubbins. Nucl. Acids Res..

[63] Carroll LM (2019). Characterization of emetic and diarrheal *Bacillus cereus* strains from a 2016 foodborne outbreak using whole-genome sequencing: addressing the microbiological, epidemiological, and bioinformatic challenges. Front. Microbiol..

[64] Nguyen LT, Schmidt HA, von Haeseler A, Minh BQ (2015). IQ-TREE: a fast and effective stochastic algorithm for estimating maximum-likelihood phylogenies. Mol. Biol. Evol..

[65] Kalyaanamoorthy S, Minh BQ, Wong TKF, von Haeseler A, Jermiin LS (2017). ModelFinder: fast model selection for accurate phylogenetic estimates. Nat. Methods.

[66] Guindon S (2010). New algorithms and methods to estimate maximum-likelihood phylogenies: assessing the performance of PhyML 3.0. Syst. Biol..

[67] Hoang DT, Chernomor O, von Haeseler A, Minh BQ, Vinh LS (2018). UFBoot2: improving the ultrafast bootstrap approximation. Mol. Biol. Evol..

[68] Rambaut A, Lam TT, Max Carvalho L, Pybus OG (2016). Exploring the temporal structure of heterochronous sequences using TempEst (formerly Path-O-Gen). Virus Evol..

[69] Bouckaert R (2014). BEAST 2: a software platform for Bayesian evolutionary analysis. PLoS Comput. Biol..

[70] Bouckaert RR, Drummond AJ (2017). bModelTest: Bayesian phylogenetic site model averaging and model comparison. BMC Evol. Biol..

[71] Drummond AJ, Ho SY, Phillips MJ, Rambaut A (2006). Relaxed phylogenetics and dating with confidence. PLoS Biol..

[72] Drummond AJ, Rambaut A, Shapiro B, Pybus OG (2005). Bayesian coalescent inference of past population dynamics from molecular sequences. Mol. Biol. Evol..

[73] Wickham H (2009). Ggplot2: Elegant Graphics for Data Analysis.

[74] Yu G, Smith DK, Zhu H, Guan Y, Lam TTY (2017). ggtree: an r package for visualization and annotation of phylogenetic trees with their covariates and other associated data. Methods Ecol. Evol..

[75] Hackathon, R. *et al.**phylobase: Base Package for Phylogenetic Structures and Comparative Data. v. 0.8.4* (2017).

[76] Yu, G. *treeio: Base Classes and Functions for Phylogenetic Tree Input and Output v. 1.4.1* (2018).

[77] Seemann T (2014). Prokka: rapid prokaryotic genome annotation. Bioinformatics.

[78] Conesa A (2005). Blast2GO: a universal tool for annotation, visualization and analysis in functional genomics research. Bioinformatics.

[79] Apweiler R (2004). UniProt: the universal protein knowledgebase. Nucl. Acids Res..

[80] Pruitt KD, Tatusova T, Maglott DR (2007). NCBI reference sequences (RefSeq): a curated non-redundant sequence database of genomes, transcripts and proteins. Nucl. Acids Res..

[81] Bauer S, Grossmann S, Vingron M, Robinson PN (2008). Ontologizer 2.0—a multifunctional tool for GO term enrichment analysis and data exploration. Bioinformatics.

[82] Grossmann S, Bauer S, Robinson PN, Vingron M (2007). Improved detection of overrepresentation of gene-ontology annotations with parent child analysis. Bioinformatics.

[83] Benjamini Y, Hochberg Y (1995). Controlling the false discovery rate: a practical and powerful approach to multiple testing. J. R. Stat. Soc. Ser. B (Methodol.).

[84] Kruskal JB (1964). Nonmetric multidimensional scaling: a numerical method. Psychometrika.

[85] Kruskal JB (1964). Multidimensional scaling by optimizing goodness of fit to a nonmetric hypothesis. Psychometrika.

[86] Chang, W., Cheng, J., Allaire, J.J., Xie, Y. & McPherson, J. *shiny: Web Application Framework for R v. 1.1.0* (2018).

[87] Oksanen, J. *et al.**vegan: Community Ecology Package v. 2.5-2* (2018).

[88] Wickham H (2011). The split-apply-combine strategy for data analysis. J. Stat. Softw..

[89] Wickham, H., François, R., Henry, L. & Müller, K. *dplyr: A Grammar of Data Manipulation v. 0.7.6* (2018).

[90] Maechler, M., Rousseeuw, P., Struyf, A., Hubert, M. & Hornik, K. *cluster: Cluster Analysis Basics and Extensions v. 2.0.7-1* (2018).

[91] Slowikowski, K. *ggrepel: Automatically Position Non-overlapping Text Labels with 'ggplot2' v. 0.8.0* (2018).

[92] Tonkin-Hill G, Lees JA, Bentley SD, Frost SDW, Corander J (2018). RhierBAPS: an R implementation of the population clustering algorithm hierBAPS. Wellcome Open Res..

[93] Cheng L, Connor TR, Siren J, Aanensen DM, Corander J (2013). Hierarchical and spatially explicit clustering of DNA sequences with BAPS software. Mol. Biol. Evol..

[94] Clarke KR (1993). Non-parametric multivariate analyses of changes in community structure. Aust. J. Ecol..

[95] Anderson MJ (2001). A new method for non-parametric multivariate analysis of variance. Austral Ecol..

[96] Anderson MJ (2006). Distance-based tests for homogeneity of multivariate dispersions. Biometrics.

[97] Anderson MJ, Walsh DCI (2013). PERMANOVA, ANOSIM, and the Mantel test in the face of heterogeneous dispersions: what null hypothesis are you testing?. Ecol. Monogr..

[98] Alikhan NF, Zhou Z, Sergeant MJ, Achtman M (2018). A genomic overview of the population structure of *Salmonella*. PLoS Genet..

[99] Treangen TJ, Ondov BD, Koren S, Phillippy AM (2014). The Harvest suite for rapid core-genome alignment and visualization of thousands of intraspecific microbial genomes. Genome Biol..

[100] Bruen TC, Philippe H, Bryant D (2006). A simple and robust statistical test for detecting the presence of recombination. Genetics.

[101] Kodama Y, Shumway M, Leinonen R, International Nucleotide Sequence Database C (2012). The sequence read archive: explosive growth of sequencing data. Nucl. Acids Res..

