# Supplementary material for: Twentieth-century emergence of antimicrobial resistant human- and bovine-associated Salmonella enterica serotype Typhimurium lineages in New York State
Source: Sci Rep. 2020 Sep 2;10:14428. doi: 10.1038/s41598-020-71344-9 (PMC7467927; doi:10.1038/s41598-020-71344-9)
Supplement: Supplementary file 1 — Supplementary Information. [file 41598_2020_71344_MOESM1_ESM.pdf]

SUPPLEMENTARY INFORMATION

Twentieth-century emergence of antimicrobial resistant human- and bovine-associated *Salmonella enterica* serotype Typhimurium lineages in New York State

Laura M. Carroll[1], Jana S. Huisman[2,3], Martin Wiedmann[1]*

[1]Department of Food Science, Cornell University, Ithaca, NY, USA

[2]Department of Environmental Systems Science, ETH Zurich, Zurich, Switzerland

[3]Swiss Institute of Bioinformatics, Lausanne, Switzerland

*Corresponding Author: Martin Wiedmann, martin.wiedmann@cornell.edu

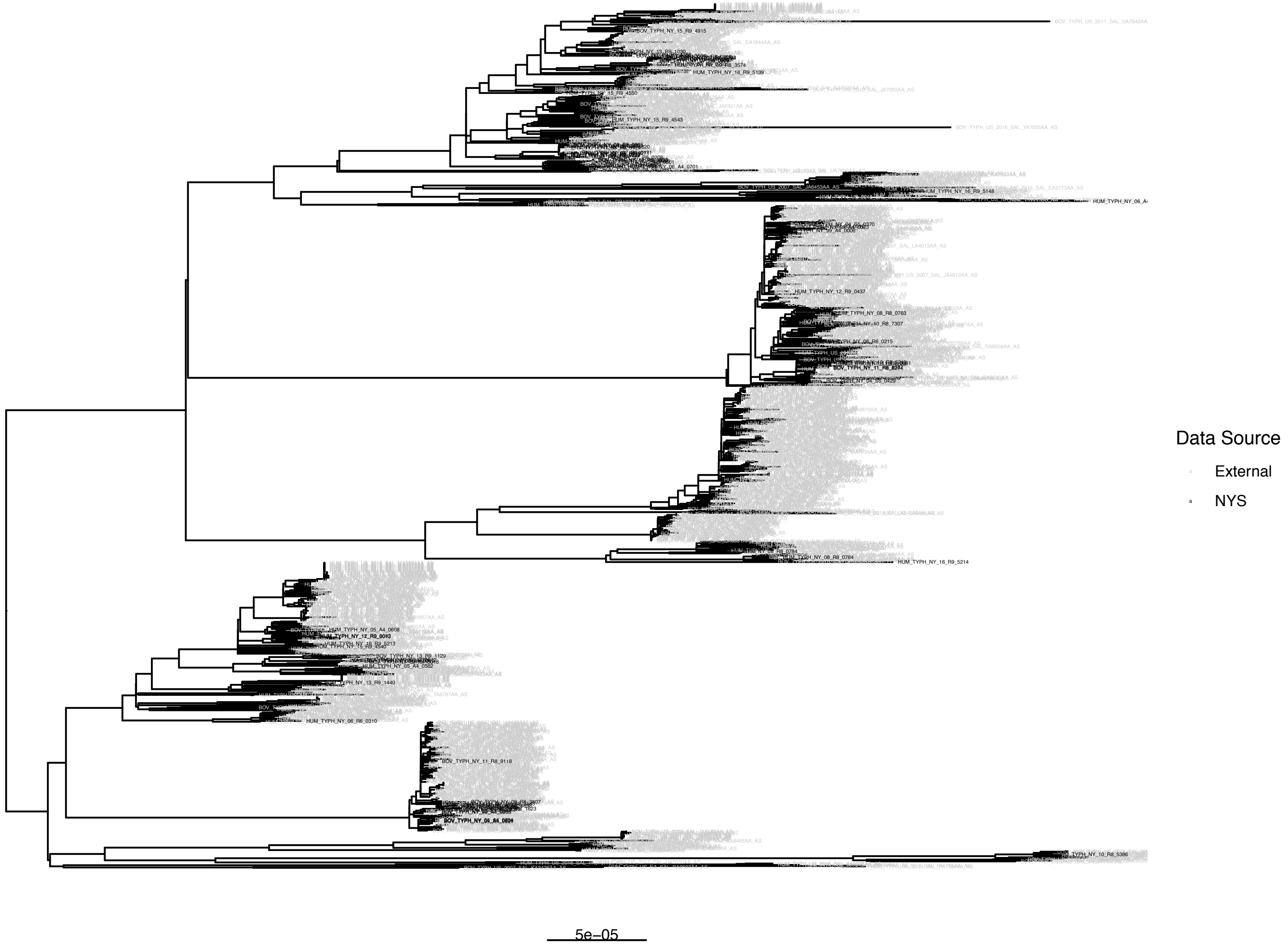

Supplementary Figure S1. Maximum likelihood phylogeny constructed using Parsnp and core SNPs identified in 1,207 bovine- and human-associated *S.* Typhimurium genomes from the United States. Tip labels in black correspond to the 87 New York state *S.* Typhimurium isolates sequenced in this study. Tip labels in gray correspond to 1,119 publicly available *S.* Typhimurium genomes downloaded via Enterobase and serotyped using SISTR (accessed November 29, 2018), as well as the reference chromosome of *S.* Typhimurium strain LT2. The phylogeny is rooted at the midpoint, and branch lengths are reported in substitutions per site.

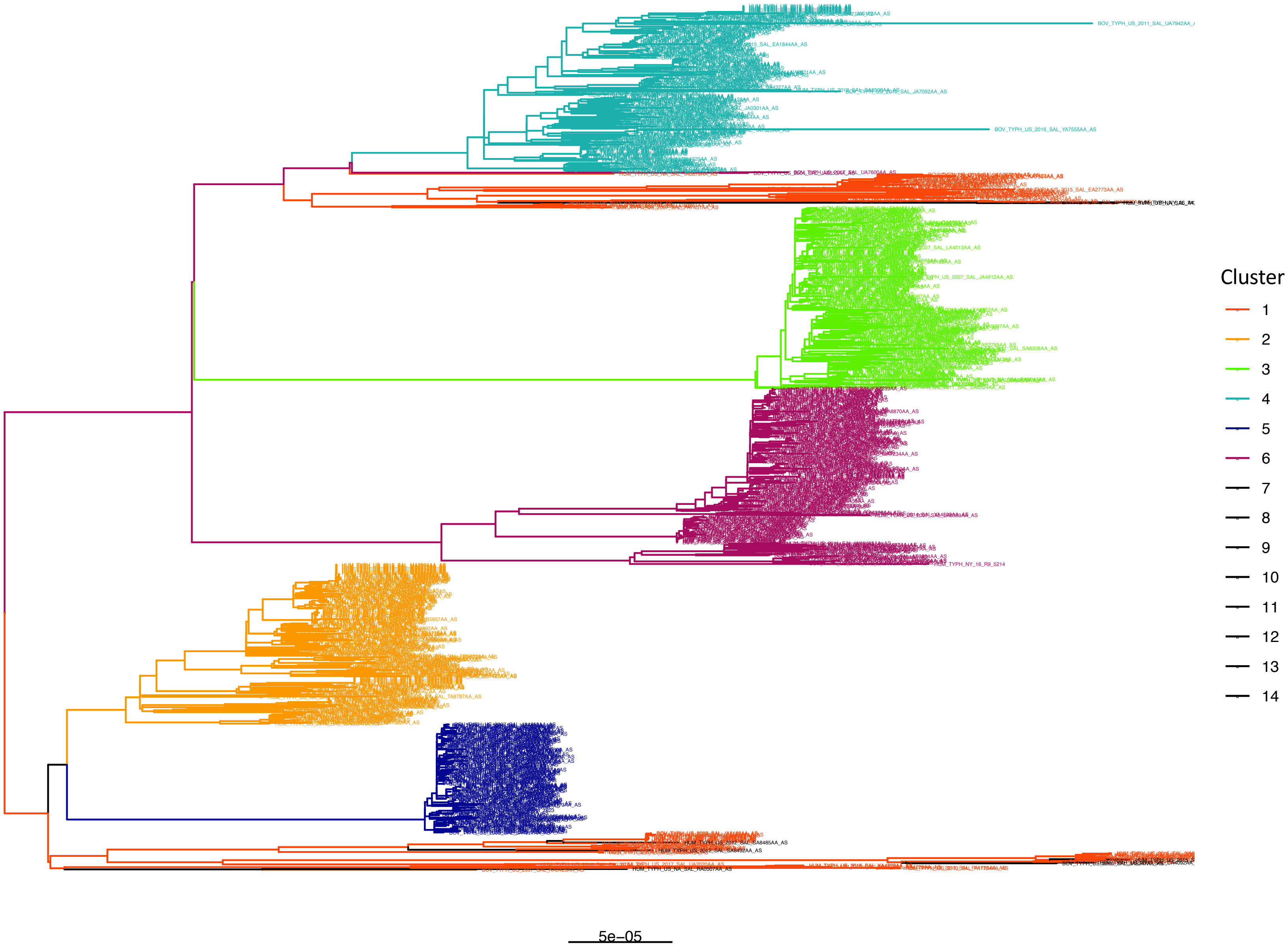

Supplementary Figure S2. Maximum likelihood phylogeny constructed using Parsnp and core SNPs identified in 1,207 bovine- and human-associated *S.* Typhimurium genomes from the United States. Tip label colors correspond to clusters assigned using rhierbaps (level 1 cluster assignments). Clusters containing only one genome (clusters 7 through 14) are shown in black. Clusters 1 (n = 92), 2 (n = 223), 3 (n = 252), 4 (n = 232), 5 (n = 152), and 6 (n = 248) are shown in color. The phylogeny is rooted at the midpoint, and branch lengths are reported in substitutions per site.

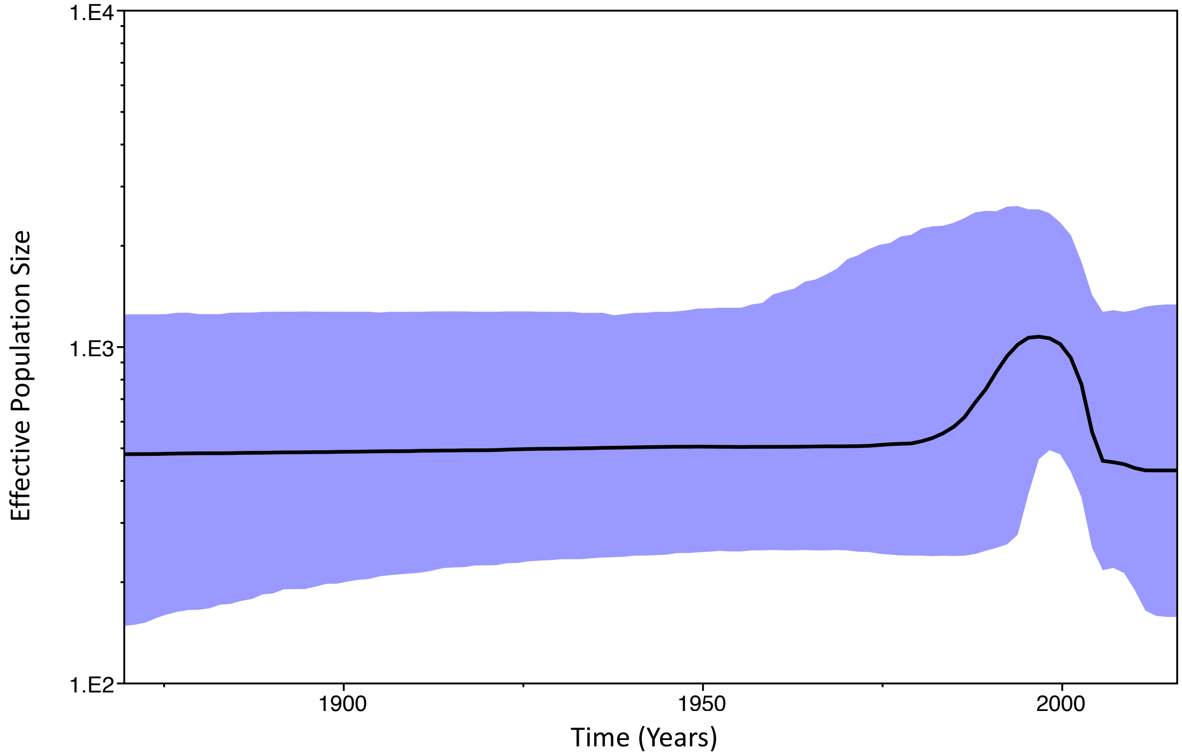

Supplementary Figure S3. Bayesian Coalescent Skyline plot constructed using 87 *S.* Typhimurium genomes from bovine, bovine farm environmental, and human clinical strains isolated in New York state from 1999 to 2016. Effective population size and time in years are plotted on the y- and x-axes, respectively. The median effective population size estimate is denoted by the black line, with its upper an the lower 95% interval estimates denoted by blue shading.

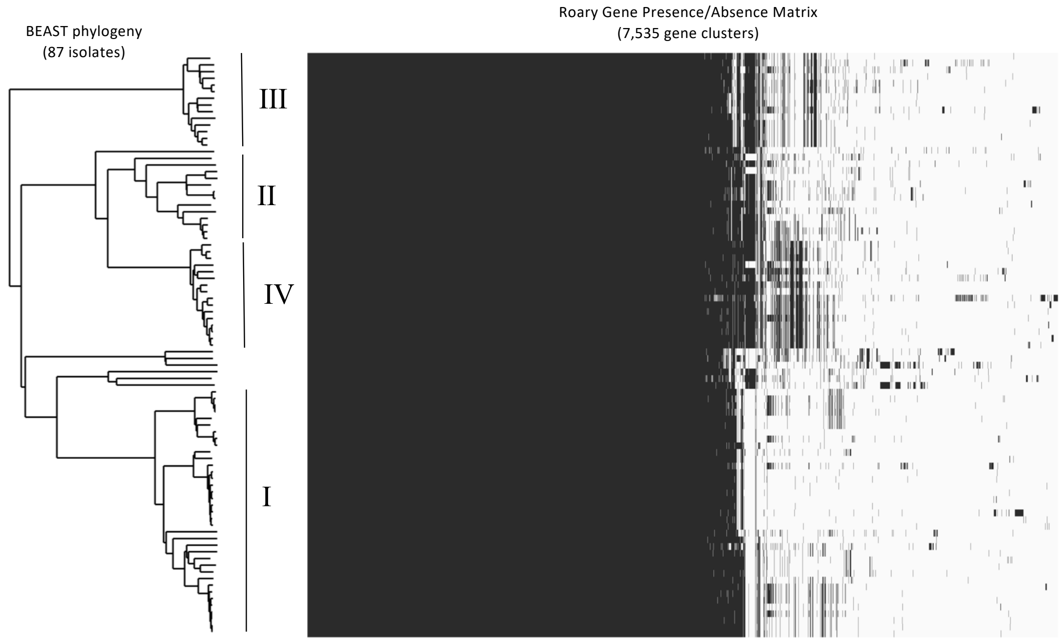

Supplementary Figure S4. Heatmap displaying the presence (black) and absence (pale gray) of 7,535 orthologous clusters detected in 87 *S.* Typhimurium isolates from New York state, 1999-2016, using Roary version 3.11.0. Each of the 87 *S.* Typhimurium isolates is represented by a tip in the phylogeny shown to the left of the heatmap, with labels representing each of the four major NYS *S.* Typhimurium lineages (I-IV) shown to the right of the phylogeny. The plot was produced using the roary_plots.py script (Marco Galardini), and the phylogeny was constructed using BEAST version 2.5.0.

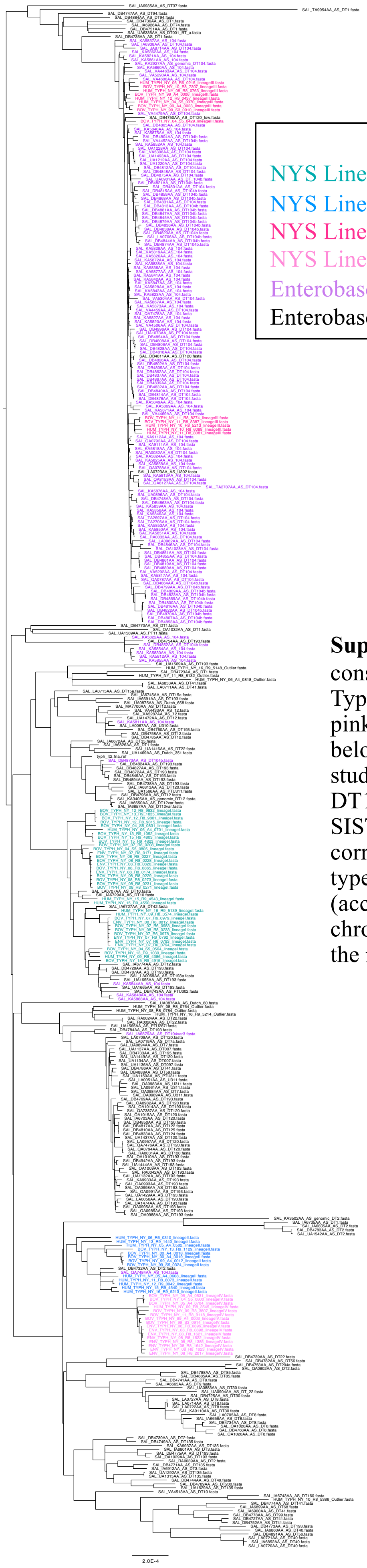

Tip Label Colors

NYS Lineage I
NYS Lineage II
NYS Lineage III
NYS Lineage IV
Enterobase *S.* Typhimurium DT104
Enterobase *S.* Typhimurium Non-DT104 Phage Types

Supplementary Figure S5. Maximum likelihood phylogeny constructed using Parsnp and core SNPs identified in 401 *S.* Typhimurium genomes. Tip labels in teal, blue, bright pink, and light pink correspond to New York state (NYS) *S.* Typhimurium isolates belonging to Lineages I, II, III, and IV, respectively, sequenced in this study (n = 87). Tip labels in purple correspond to *S.* Typhimurium DT104 genomes downloaded via Enterobase and serotyped using SISTR (accessed February 10, 2019; n = 148). Tip labels in black correspond to *S.* Typhimurium genomes belonging to other phage types, downloaded via Enterobase and serotyped using SISTR (accessed February 10, 2019; n = 165), as well as the reference chromosome of *S.* Typhimurium strain LT2. The phylogeny is rooted at the midpoint, and branch lengths are reported in substitutions per site.

Supplementary Table S1. Isolates used in this study.

Study ID	Isolation Source	Isolation Year	FMT ID[a]	Average Assembly Coverage	Assembly N50	Number of Contigs
BOV_TYPH_NY_00_A4_0018	Bovine	2000	FSL A4-0018	38.637	376792	61
BOV_TYPH_NY_00_A4_0019	Bovine	2000	FSL A4-0019	61.3141	376792	67
BOV_TYPH_NY_04_S5_0429	Bovine	2004	FSL S5-0429	35.8611	225812	84
BOV_TYPH_NY_04_S5_0564	Bovine	2004	FSL S5-0564	41.6215	207707	81
BOV_TYPH_NY_04_S5_0800	Bovine	2004	FSL S5-0800	39.1868	224303	91
BOV_TYPH_NY_04_S5_0805	Bovine	2004	FSL S5-0805	33.7295	207707	73
BOV_TYPH_NY_04_S5_0831	Bovine	2004	FSL S5-0831	36.8626	207707	77
BOV_TYPH_NY_05_A4_0531	Bovine	2005	FSL A4-0531	37.1289	213473	97
BOV_TYPH_NY_05_A4_0704	Bovine	2005	FSL A4-0704	39.8912	224303	102
BOV_TYPH_NY_07_R6_0978	Bovine	2007	FSL R6-0978	125.807	266896	79
BOV_TYPH_NY_07_R6_0979	Bovine	2007	FSL R6-0979	70.3424	266898	74
BOV_TYPH_NY_07_R6_0983	Bovine	2007	FSL R6-0983	72.6573	191686	80
BOV_TYPH_NY_07_R8_0208	Bovine	2007	FSL R8-0208	115.36	275470	70
BOV_TYPH_NY_08_R8_0227	Bovine	2008	FSL R8-0227	68.5241	275470	69
BOV_TYPH_NY_08_R8_0228	Bovine	2008	FSL R8-0228	120.336	275470	69
BOV_TYPH_NY_08_R8_0229	Bovine	2008	FSL R8-0229	99.929	188083	472
BOV_TYPH_NY_08_R8_0231	Bovine	2008	FSL R8-0231	101.261	188083	69
BOV_TYPH_NY_08_R8_0233	Bovine	2008	FSL R8-0233	59.2982	266901	90
BOV_TYPH_NY_08_R8_0271	Bovine	2008	FSL R8-0271	53.7531	275470	78
BOV_TYPH_NY_08_R8_0273	Bovine	2008	FSL R8-0273	162.685	275470	106
BOV_TYPH_NY_08_R8_0865	Bovine	2008	FSL R8-0865	57.0781	124016	105
BOV_TYPH_NY_09_R8_3807	Bovine	2009	FSL R8-3807	33.7961	253158	113
BOV_TYPH_NY_10_R8_7307	Bovine	2010	FSL R8-7307	54.94	141262	95
BOV_TYPH_NY_11_R8_8274	Bovine	2011	FSL R8-8274	57.8093	149313	135
BOV_TYPH_NY_11_R8_8387	Bovine	2011	FSL R8-8387	50.3129	124027	120

BOV_TYPH_NY_11_R8_9118	Bovine	2011	FSL R8-9118	68.2967	191779	121
BOV_TYPH_NY_12_R8_9801	Bovine	2012	FSL R8-9801	90.3807	125611	120
BOV_TYPH_NY_12_R8_9815	Bovine	2012	FSL R8-9815	72.5053	101023	114
BOV_TYPH_NY_12_R8_9832	Bovine	2012	FSL R8-9832	70.4658	118805	120
BOV_TYPH_NY_13_R9_1030	Bovine	2013	FSL R9-1030	35.8702	191771	83
BOV_TYPH_NY_13_R9_1052	Bovine	2013	FSL R9-1052	45.7753	207707	71
BOV_TYPH_NY_13_R9_1129	Bovine	2013	FSL R9-1129	33.0865	249596	60
BOV_TYPH_NY_13_R9_1835	Bovine	2013	FSL R9-1835	58.0566	207707	64
BOV_TYPH_NY_15_R9_4803	Bovine	2015	FSL R9-4803	36.8403	207707	79
BOV_TYPH_NY_15_R9_4823	Bovine	2015	FSL R9-4823	40.3555	275479	70
BOV_TYPH_NY_15_R9_4915	Bovine	2015	FSL R9-4915	31.5239	189572	84
BOV_TYPH_NY_99_A4_0003	Bovine	1999	FSL A4-0003	33.3913	224302	92
BOV_TYPH_NY_99_A4_0006	Bovine	1999	FSL A4-0006	36.2804	204653	96
BOV_TYPH_NY_99_A4_0012	Bovine	1999	FSL A4-0012	33.6407	225813	74
BOV_TYPH_NY_99_A4_0023	Bovine	1999	FSL A4-0023	29.3371	180514	100
BOV_TYPH_NY_99_S3_0910	Bovine	1999	FSL S3-0910	45.9742	223066	84
BOV_TYPH_NY_99_S3_0914	Bovine	1999	FSL S3-0914	47.1549	271001	77
BOV_TYPH_NY_99_S5_0324	Bovine	1999	FSL S5-0324	40.2861	376792	66
ENV_TYPH_NY_07_R6_0792	Farm environment	2007	FSL R6-0792	87.3533	266901	85
ENV_TYPH_NY_07_R6_0793	Farm environment	2007	FSL R6-0793	104.018	266896	100
ENV_TYPH_NY_07_R6_0794	Farm environment	2007	FSL R6-0794	56.8704	191686	81
ENV_TYPH_NY_07_R8_0171	Farm environment	2007	FSL R8-0171	92.1302	275470	82
ENV_TYPH_NY_08_R8_0174	Farm environment	2008	FSL R8-0174	79.0213	275470	85
ENV_TYPH_NY_08_R8_0812	Farm environment	2008	FSL R8-0812	80.9941	207864	138
ENV_TYPH_NY_08_R8_0820	Farm environment	2008	FSL R8-0820	87.3184	184443	103
ENV_TYPH_NY_08_R8_0896	Farm environment	2008	FSL R8-0896	105.197	225890	136
ENV_TYPH_NY_08_R8_0898	Farm environment	2008	FSL R8-0898	66.889	176925	135
ENV_TYPH_NY_08_R8_1385	Farm environment	2008	FSL R8-1385	108.032	33489	390
ENV_TYPH_NY_08_R8_1621	Farm environment	2008	FSL R8-1621	60.1931	225826	202

ENV_TYPH_NY_08_R8_1622	Farm environment	2008	FSL R8-1622	119.063	225827	167
ENV_TYPH_NY_08_R8_1623	Farm environment	2008	FSL R8-1623	32.596	224130	139
ENV_TYPH_NY_08_R8_1642	Farm environment	2008	FSL R8-1642	69.3444	219324	115
ENV_TYPH_NY_08_R8_2017	Farm environment	2008	FSL R8-2017	78.5042	224302	103
HUM_TYPH_NY_04_S5_0370	Human clinical	2004	FSL S5-0370	39.7194	223066	83
HUM_TYPH_NY_05_A4_0582	Human clinical	2005	FSL A4-0582	37.1961	275599	56
HUM_TYPH_NY_05_A4_0608	Human clinical	2005	FSL A4-0608	42.8189	271390	63
HUM_TYPH_NY_06_A4_0701	Human clinical	2006	FSL A4-0701	36.0825	191681	68
HUM_TYPH_NY_06_A4_0818	Human clinical	2006	FSL A4-0818	47.406	280700	65
HUM_TYPH_NY_06_R6_0215	Human clinical	2006	FSL R6-0215	38.248	223068	61
HUM_TYPH_NY_06_R6_0310	Human clinical	2006	FSL R6-0310	30.544	271390	80
HUM_TYPH_NY_08_R8_0763	Human clinical	2008	FSL R8-0763	67.7583	123409	105
HUM_TYPH_NY_08_R8_0764	Human clinical	2008	FSL R8-0764	46.2547	160163	103
HUM_TYPH_NY_08_R8_0784	Human clinical	2008	FSL R8-0784	77.3604	164269	90
HUM_TYPH_NY_09_R8_3545	Human clinical	2009	FSL R8-3545	35.5299	271001	67
HUM_TYPH_NY_09_R8_3574	Human clinical	2009	FSL R8-3574	39.1837	207869	84
HUM_TYPH_NY_09_R8_4386	Human clinical	2009	FSL R8-4386	33.8281	201851	84
HUM_TYPH_NY_10_R8_5213	Human clinical	2010	FSL R8-5213	75.9884	105799	114
HUM_TYPH_NY_10_R8_5386	Human clinical	2010	FSL R8-5386	30.2035	223675	73
HUM_TYPH_NY_10_R8_6089	Human clinical	2010	FSL R8-6089	69.6223	113216	126
HUM_TYPH_NY_11_R8_8073	Human clinical	2011	FSL R8-8073	78.6398	168875	119
HUM_TYPH_NY_11_R8_8081	Human clinical	2011	FSL R8-8081	81.0544	148849	125
HUM_TYPH_NY_11_R8_8132	Human clinical	2011	FSL R8-8132	78.9084	172389	120
HUM_TYPH_NY_12_R9_0042	Human clinical	2012	FSL R9-0042	65.7272	108722	120
HUM_TYPH_NY_12_R9_0437	Human clinical	2012	FSL R9-0437	53.5624	128384	110
HUM_TYPH_NY_13_R9_1440	Human clinical	2013	FSL R9-1440	35.3403	271390	65
HUM_TYPH_NY_15_R9_4540	Human clinical	2015	FSL R9-4540	36.8146	225812	67
HUM_TYPH_NY_15_R9_4543	Human clinical	2015	FSL R9-4543	31.692	165253	102
HUM_TYPH_NY_15_R9_4550	Human clinical	2015	FSL R9-4550	29.3771	187898	87

HUM_TYPH_NY_16_R9_5139	Human clinical	2016	FSL R9-5139	32.8499	162636	129
HUM_TYPH_NY_16_R9_5148	Human clinical	2016	FSL R9-5148	38.5758	278004	63
HUM_TYPH_NY_16_R9_5213	Human clinical	2016	FSL R9-5213	44.4612	376793	64
HUM_TYPH_NY_16_R9_5214	Human clinical	2016	FSL R9-5214	34.2787	282609	80

[a]Food Microbe Tracker ID (http://www.foodmicrobetracker.com)

Supplementary Table S2. Gene ontology (GO) terms over-represented among four *S.* Typhimurium lineages.

**Lineage**[a]	**GO ID**[b]	**GO Class**[c]	**GO Name**[d]	**BH-Adjusted *P*-Value**[e]
I	GO:0042710	Biological Process	biofilm formation	5.19E-13
I	GO:0048870	Biological Process	cell motility	0.001118274
I	GO:0090092	Biological Process	regulation of transmembrane receptor protein serine/threonine kinase signaling pathway	0.009649429
I	GO:0090101	Biological Process	negative regulation of transmembrane receptor protein serine/threonine kinase signaling pathway	0.009649429
I	GO:0044464	Cellular Component	cell part	0.009649429
I	GO:0005623	Cellular Component	cell	0.009649429
II	GO:0009753	Biological Process	response to jasmonic acid	0.039397537
II	GO:0000746	Biological Process	conjugation	0.039397537
III	GO:0044433	Cellular Component	cytoplasmic vesicle part	5.40E-08
III	GO:0043473	Biological Process	pigmentation	3.42E-07
III	GO:0000746	Biological Process	conjugation	3.70E-07
III	GO:0043150	Biological Process	DNA synthesis involved in double-strand break repair via homologous recombination	4.02E-05
III	GO:0015655	Molecular Function	alanine:sodium symporter activity	0.000759039
III	GO:0018801	Molecular Function	glutaconyl-CoA decarboxylase activity	0.017266619
III	GO:0015505	Molecular Function	uracil:cation symporter activity	0.049752896
IV	GO:0052192	Biological Process	movement in environment of other organism involved in symbiotic interaction	2.83E-54
IV	GO:0019012	Cellular Component	virion	2.54E-48
IV	GO:0051701	Biological Process	interaction with host	4.92E-37
IV	GO:0019069	Biological Process	viral capsid assembly	1.23E-26
IV	GO:0044403	Biological Process	symbiont process	7.14E-24
IV	GO:0019076	Biological Process	viral release from host cell	1.14E-23
IV	GO:0051704	Biological Process	multi-organism process	3.88E-20
IV	GO:0016032	Biological Process	viral process	1.62E-17
IV	GO:0052126	Biological Process	movement in host environment	2.55E-17

IV	GO:0015266	Molecular Function	protein channel activity	7.68E-17
IV	GO:0034290	Molecular Function	holin activity	9.66E-17
IV	GO:0019835	Biological Process	cytolysis	1.73E-13
IV	GO:0044215	Cellular Component	other organism	3.52E-13
IV	GO:0044217	Cellular Component	other organism part	3.52E-13
IV	GO:0051673	Biological Process	membrane disruption in other organism	4.38E-13
IV	GO:0040011	Biological Process	locomotion	7.14E-13
IV	GO:0032196	Biological Process	transposition	2.91E-11
IV	GO:0044423	Cellular Component	virion part	3.04E-11
IV	GO:0045340	Molecular Function	mercury ion binding	5.24E-11
IV	GO:0046812	Molecular Function	host cell surface binding	7.61E-11
IV	GO:0015694	Biological Process	mercury ion transport	2.45E-10
IV	GO:0015097	Molecular Function	mercury ion transmembrane transporter activity	2.97E-10
IV	GO:0047066	Molecular Function	phospholipid-hydroperoxide glutathione peroxidase activity	4.16E-10
IV	GO:0031312	Cellular Component	extrinsic component of organelle membrane	6.11E-10
IV	GO:0080167	Biological Process	response to karrikin	6.54E-10
IV	GO:0044660	Biological Process	cytolysis by virus via pore formation in host cell membrane	6.63E-10
IV	GO:0031314	Cellular Component	extrinsic component of mitochondrial inner membrane	7.04E-10
IV	GO:0000746	Biological Process	conjugation	8.35E-10
IV	GO:0031315	Cellular Component	extrinsic component of mitochondrial outer membrane	1.15E-09
IV	GO:0044419	Biological Process	interspecies interaction between organisms	3.05E-09
IV	GO:0015074	Biological Process	DNA integration	4.12E-09
IV	GO:0006842	Biological Process	tricarboxylic acid transport	1.18E-08
IV	GO:0019271	Biological Process	aerobactin transport	1.33E-08
IV	GO:0098025	Cellular Component	virus tail, baseplate	1.57E-08
IV	GO:0019058	Biological Process	viral life cycle	6.20E-08
IV	GO:0046790	Molecular Function	virion binding	1.02E-07
IV	GO:0046689	Biological Process	response to mercury ion	2.15E-07
IV	GO:0004803	Molecular Function	transposase activity	3.28E-07

IV	GO:0006310	Biological Process	DNA recombination	6.07E-07
IV	GO:0052111	Biological Process	modification by symbiont of host structure	9.51E-07
IV	GO:0052185	Biological Process	modification of structure of other organism involved in symbiotic interaction	9.51E-07
IV	GO:0035890	Biological Process	exit from host	1.65E-06
IV	GO:0044657	Biological Process	pore formation in membrane of other organism during symbiotic interaction	4.03E-06
IV	GO:0016443	Molecular Function	bidentate ribonuclease III activity	4.58E-06
IV	GO:0044406	Biological Process	adhesion of symbiont to host	4.73E-06
IV	GO:0006259	Biological Process	DNA metabolic process	1.23E-05
IV	GO:0009214	Biological Process	cyclic nucleotide catabolic process	0.000163889
IV	GO:0050787	Biological Process	detoxification of mercury ion	0.000188513
IV	GO:0140097	Molecular Function	catalytic activity, acting on DNA	0.000280597
IV	GO:0016152	Molecular Function	mercury (II) reductase activity	0.000697487
IV	GO:0000150	Molecular Function	recombinase activity	0.000697487
IV	GO:0020002	Cellular Component	host cell plasma membrane	0.001026026
IV	GO:0034071	Molecular Function	aminoglycoside phosphotransferase activity	0.001953707
IV	GO:0003796	Molecular Function	lysozyme activity	0.003438745
IV	GO:0090304	Biological Process	nucleic acid metabolic process	0.004309037
IV	GO:0051002	Molecular Function	ligase activity, forming nitrogen-metal bonds	0.00472909
IV	GO:0003676	Molecular Function	nucleic acid binding	0.008099171
IV	GO:0039633	Biological Process	killing by virus of host cell	0.015841967
IV	GO:0004156	Molecular Function	dihydropteroate synthase activity	0.015841967
IV	GO:0006139	Biological Process	nucleobase-containing compound metabolic process	0.021568623
IV	GO:0031968	Cellular Component	organelle outer membrane	0.02382464
IV	GO:0061687	Biological Process	detoxification of inorganic compound	0.024982796
IV	GO:0016998	Biological Process	cell wall macromolecule catabolic process	0.024982796
IV	GO:0006313	Biological Process	transposition, DNA-mediated	0.025617526
IV	GO:0001906	Biological Process	cell killing	0.030479767
IV	GO:0005741	Cellular Component	mitochondrial outer membrane	0.038472528

[a]Lineages I (n = 37), II (n = 13), III (n = 14), and IV (n = 16)

[b]Gene Ontology (GO) identifier (ID)
[c]Gene Ontology (GO) class; can be one of "Biological Process", "Cellular Component", or "Molecular Function"
[d]Gene Ontology (GO) name
[e]*P*-values obtained using Ontologizer version 2.1 and adjusted to control for the false discovery rate (FDR) at the 0.05 level using the Benjamini-Hochberg procedure

Supplementary Table S3. Virulence factors detected in all 87 *S.* Typhimurium genomes from New York state.[a]

avrA	*invC*	*pipB*	*sopD*	*ssaO*	*sseK2*
csgA	*invE*	*pipB2*	*sopD2*	*ssaP*	*sseL*
csgB	*invF*	*prgH*	*sopE2*	*ssaQ*	*sspH2*
csgC	*invG*	*prgI*	*spaO*	*ssaR*	*steA*
csgD	*invH*	*prgJ*	*spaP*	*ssaS*	*steB*
csgE	*invI*	*prgK*	*spaQ*	*ssaT*	*steC*
csgF	*invJ*	*ratB*	*spaR*	*ssaU*	
csgG	*lpfA*	*sicA*	*spaS*	*ssaV*	
entA	*lpfB*	*sicP*	*spiC/ssaB*	*sscA*	
entB	*lpfC*	*sifA*	*sptP*	*sscB*	
fepC	*lpfD*	*sifB*	*ssaC*	*sseA*	
fepG	*lpfE*	*sinH*	*ssaD*	*sseB*	
fimC	*mgtB*	*sipA/sspA*	*ssaE*	*sseC*	
fimD	*mgtC*	*sipB/sspB*	*ssaG*	*sseD*	
fimF	*mig-14*	*sipC/sspC*	*ssaH*	*sseE*	
fimH	*misL*	*sipD*	*ssaI*	*sseF*	
fimI	*ompA*	*slrP*	*ssaJ*	*sseG*	
fliN	*orgA*	*sodCI*	*ssaK*	*sseI/srfH*	
invA	*orgB*	*sopA*	*ssaL*	*sseJ*	
invB	*orgC*	*sopB/sigD*	*ssaM*	*sseK1*	

[a]Virulence factors were detected in all genomes using ABRicate version 0.8 and the Virulence Factors Database (VFDB; June 11, 2018), using minimum identity and coverage thresholds of 80 and 50%, respectively.
